# Effects of different types of modifiers on structural variation of nano-hydroxyapatite for efficient application

**DOI:** 10.1039/d5na00392j

**Published:** 2025-06-24

**Authors:** Nahida Sultana Bristy, Md. Kawsar, Md. Sahadat Hossain

**Affiliations:** a Department of Chemical Engineering, Bangladesh University of Engineering and Technology Dhaka Bangladesh; b Department of Applied Chemistry and Chemical Engineering, Noakhali Science and Technology University Noakhali Bangladesh Kawsar.acce@gmail.com; c Glass Research Division, Institute of Glass & Ceramic Research and Testing, Bangladesh Council of Scientific and Industrial Research (BCSIR) Dhaka 1205 Bangladesh saz8455@gmail.com

## Abstract

Hydroxyapatite (HAp) has emerged as a biomaterial of significant interest due to its intrinsic biocompatibility and structural similarity to natural bone minerals. While HAp is traditionally derived from natural sources, chemical synthesis *via* conventional methods, such as wet chemical precipitation and sol–gel processing, and newer techniques like microwave-assisted synthesis and hydrothermal methods have enabled greater control over its physicochemical properties. With the expansion of applications beyond conventional biomedical uses, recent research has concentrated on engineering nanohydroxyapatite with precisely tailored morphologies and structures. This review examines the influence of various organic modifiers on nano-HAp synthesis, highlighting how these agents modulate its crystal growth, crystallinity, surface topology, particle dimensions, and porosity. Potent chelating agents (*e.g.*, citric acid and EDTA) have been shown to yield purer, more uniform nanoparticles, whereas cationic–anionic surfactants (*e.g.*, CTAB and SDS) enhance the surface area. Modifiers such as Triton X-100, chitosan, and polyethylene glycol effectively adjust the pore size. Scientists are also investigating environmentally friendly and toxicant-free modifiers. Through summarization of insights from current literature, this review provides a comprehensive framework for selecting suitable modifiers to fabricate well-defined HAp nanomaterials for diverse applications in future studies.

## Abstract 2

Structural modification of hydroxyapatites for fruitful applications in various fields.

## Graphical abstract

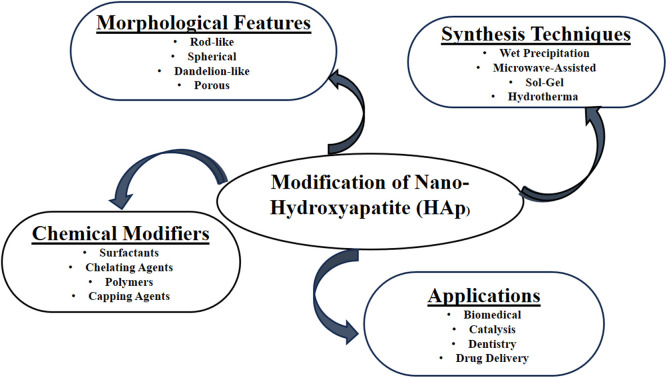

## 1. Introduction

**Fig. 1:**
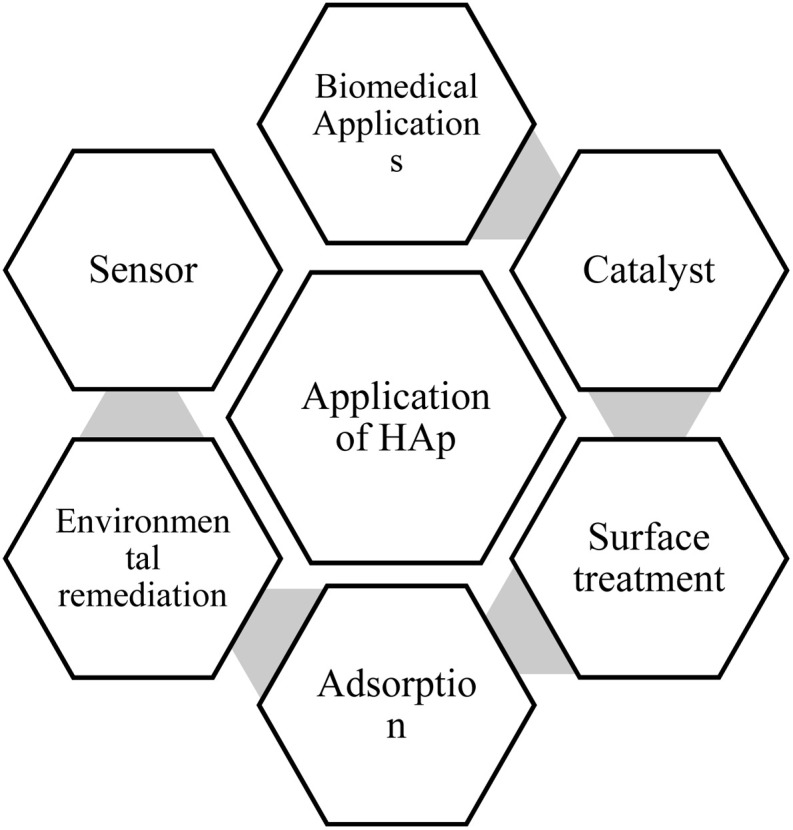
Application of HAp in different fields.

In the current era of advanced materials research, hydroxyapatite (HAp) has become one of the most widely investigated biomaterials as it spans multiple disciplines, including medicine,^1^ dentistry,^2^ agriculture,^3^ industrial fields,^4^ environmental science,^5^*etc.* Different application fields of HAp are visualized (Fig. 1)*.* HAp with the chemical formula Ca_10_(PO)_6_(OH)_2_ closely resembles the inorganic component of bones and teeth.^6–9^ Its exceptional properties, such as biocompatibility and reactivity, make it ideal for bone tissue engineering, drug delivery, and orthopedic applications.^10–12^ The term “apatite” was first used by Werner in 1788 to refer to a family of compounds with similar hexagonal crystal structures and space groups despite varying compositions. After the development of X-ray diffraction, Dejong in 1926 confirmed that apatite is identical to the mineral component of bones and teeth.^13,14^ Among the significant apatite groups, HAp has been extensively studied since the 1950s for its usage in medical disciplines.^15,16^ Apart from medical usage, HAp also became worthwhile for industrial and technological applications such as a catalyst in chemical reactions,^17^ a host material for lasers,^18^ fluorescence materials,^19^ ion conductors,^20^ and gas sensors.^21^ Furthermore, synthetic HAp is employed in protein and nucleic acid fractionation *via* column chromatography^22^ and water treatment^23^ and soil remediation^24^ for heavy metal contamination.^25^ Hydroxyapatite (HAp) continues to be a focal point of scientific research. For instance, polycaprolactone/nano-hydroxyapatite (PCL/nano-HAp) nanocomposites have been utilized to fabricate drug-loaded implants through solution-extrusion 3D printing which have superior mechanical properties;^26^ recently developed carboxymethylcellulose–Al(iii)/HAp aerogel beads are capable of selectively removing fluoride from brick tea infusions without altering sensory properties, achieving adsorption capacities over 23 mg g^−1^;^27^ gold/hydroxyapatite nanocomposites functionalized with polydopamine nanocomposites modulate immune responses and facilitate vascularized bone regeneration;^28^ collagen and κ-carrageenan fabricated with hydroxyapatite reinforced with lanthanum oxide nanoparticles, a biocomposite, has been shown to speed up the bone repair process.^29^ These recent studies highlight that researchers are actively exploring new ways to improve hydroxyapatite.

HAp has a Ca/P molar ratio of 1.67, which is the right balance with high stability of HAp and good mechanical properties.^30,31^ Primarily, it exists in two nano-crystalline structures—hexagonal phase (*P*6_3_/*m* or *P*6_3_) and monoclinic phase (*P*2_1_/*b* or *P*2_1_).^32^ In hexagonal HAp, OH groups are aligned along the *c*-axis in two order variations: hexagonal disordered *P*6_3_/*m*, where OH dipoles are randomly distributed over the whole crystal between the neighboring unit cells, and ordered *P*6_3_ with a parallel orientation, a common phase found in synthetic hydroxyapatite. In monoclinic HAp, they also have the same variations as the hexagonal one but are less common.^33^ The properties of hydroxyapatite mostly depend on its preparation method or origin. Studies showed that HAp from biological sources exhibits higher crystallinity (at 800 °C) than synthetic HAp, whereas the synthetic one has a larger surface area and porosity.^34^ With the evolution of nanotechnology, nanosized HAp gained significant attention due to its improved qualities compared to normal HAp.^35^ Superior biological responses such as bone regeneration, osteoblast adhesion, and proliferation made nano-HAp highly valuable.^36^ Several research studies have been conducted to study the characteristics of the nanoparticles of HAp. Recent investigations revealed that nano-HAp has a complex surface structure, and the nanoparticles consist of a crystalline core that is elongated along the crystallographic *c*-axis.^37^ Furthermore, it is assumed that the nanocrystals have a grain size of less than 100 nm in at least one direction, closely resembling the mineral found in hard tissues.^38^ Conventional HAp cannot withstand high loads and is prone to brittle failure.^39^ Nano-HAp follows the Hall–Petch relationship,^40^ where the strength of the material increases with decreasing grain size.^41^ Furthermore, nano-HAp possesses higher dissolution rates^42^ because of the increased grain boundaries.^43^ Among various morphologies of nano-HAp, needle-like and spherical shapes are the most common and applicable.^44^ Owing to the exceptional physical and chemical properties of HAp, many synthesis methods have been developed by scientists to modify its morphologies, sizes, crystallinity, calcium–phosphate ratio, and other characteristics for specific applications.^45,46^ These synthesis methods are significantly influenced by reaction conditions (reaction temperature, pH, calcination temperature, time, initial concentration, *etc.*).^47^ For tailored applications, modifiers have received much recognition for the synthesis of nano-HAp with controlled properties, especially organic modifiers are extensively used such as citric acid-mediated F-doped mesoporous HAp, which has biocidal implant application,^48^ surface-modified HAp with stearic acid (SA) is used as a coating agent for titanium dental implants.^49^ Modifiers including urea, fatty acids, amino acids, citric acid,^50^ carboxylic acids, cetyltrimethylammonium bromide (CTAB), sodium dodecyl sulfate (SDS),^51^ ethylenediaminetetraacetic acid (EDTA), Tween 20, trisodium citrate, and d-sorbitol^52^ are successfully used in different processes for controlled synthesis.^53,54^

While many studies have explored how reaction conditions affect HAp's structure, there is still little research on how modifiers influence its properties, to the best of our knowledge. This review provides a comprehensive analysis of the existing literature on modifiers used for the structural variation of nano-hydroxyapatite (nano-HAp), aiming to facilitate future investigations to fill the knowledge gap. For this particular review paper, we will be discussing the effect of modifiers on the structural variation of nano-HAp synthesized by some of the most significant synthesis methods: the wet chemical technique, microwave-assisted method, sol–gel method, and hydrothermal method for efficient uses.

## 2. Synthesis methods

Several methods have been developed for synthesizing hydroxyapatite (HAp), and each of these methods results in unique characteristics of HAp.^55^ These techniques can be classified into dry (*e.g.*, solid-state synthesis and mechanochemical method), wet (*e.g.*, wet precipitation and sol–gel), and high-temperature methods (*e.g.*, combustion and pyrolysis).^56^ Among these, the most commonly employed approaches, wet chemical, sol–gel, hydrothermal, and microwave-assisted methods, will be discussed here (Table 1).

**Table 1:** Different synthesis methods for HAp^54^

Dry method	• Solid-state synthesis
	• Mechanochemical method
Wet method	• Wet precipitation
	• Sol–gel
	• Hydrolysis
	• Hydrothermal
	• Emulsion
	• Sonochemical
High-temperature method	• Combustion
	• Pyrolysis

### 2.1 Wet chemical method

**Fig. 2:**
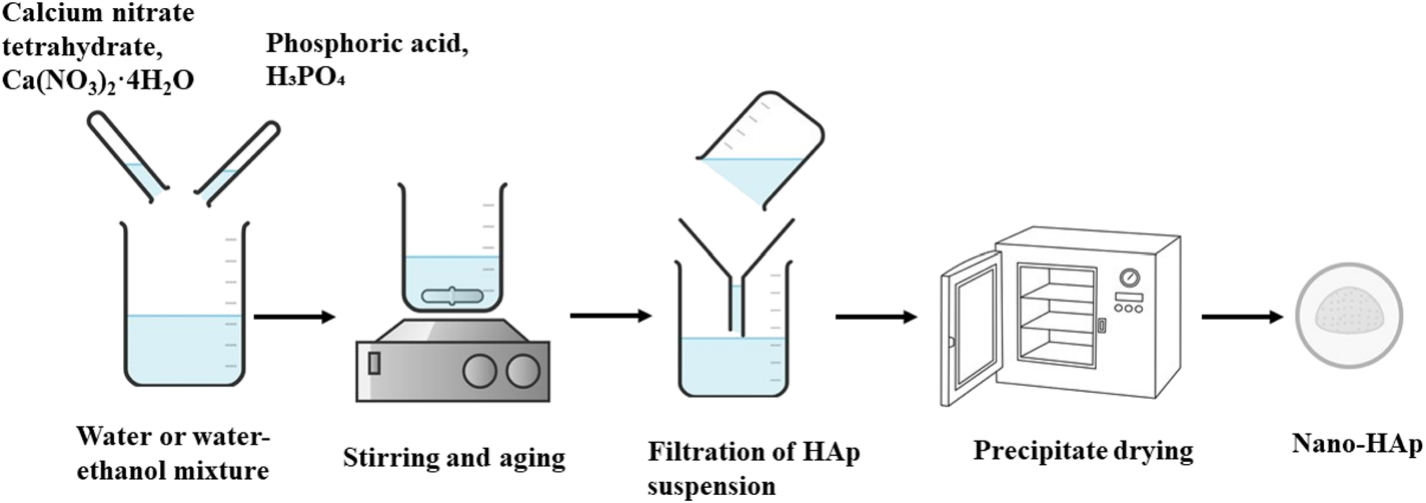
Synthesis of hydroxyapatite by the wet chemical method.

In 1976, Jarcho and his colleagues first explored the wet-chemical precipitation method to produce a dense polycrystalline hydroxyapatite with high mechanical properties. Since then, many researchers have refined and expanded this technique.^57^ Common sources of calcium for the wet chemical method are calcium hydroxide (Ca(OH)_2_), calcium nitrate tetrahydrate (Ca(NO_3_)_2_·4H_2_O), and calcium oxide (CaO) and sources of phosphorus are phosphoric acid (H_3_PO_4_) and diammonium hydrogen phosphate ((NH_4_)_2_HPO_4_). For the synthesis, reactants are dissolved in water or a water–ethanol mixture, then stirred and aged at room temperature to 85 °C overnight, with the pH kept at 9–11. The filtered precipitate is dried using atmospheric drying, vacuum drying, or freeze drying and calcined at temperatures between 700 °C and 1250 °C.^58–61^ Several characterization techniques, such as X-ray diffraction (XRD), Scanning Electron Microscopy (SEM), Fourier Transform Infrared (FTIR) spectroscopy, Transmission Electron Microscopy (TEM), Differential Thermal Analysis (DTA), and chemical analysis – Atomic Absorption Spectroscopy (AAS) or EDTA titration, demonstrated the purity (nearly pure) with a low level of impurity content.^62^ Other benefits, such as the minimal processing temperature and the ability to produce highly intricate nanomaterials and adapt according to specific applications, have drawn the attention of researchers.^63^ Studies have shown that altering the critical processing parameters, such as temperature, pH, concentration of reactants, and aging time, can modify the physicochemical properties (morphology, particle size, and crystallinity), which have a significant influence on the biological response and clinical performance of nano-HAp.^64^ For instance, spherical-shaped HAp nanoparticles with smaller particle sizes (21–78 nm) can be obtained under alkaline conditions (pH 11), while neutral to moderately basic pH conditions give particles shaped as beaded rods, nanorods, nanoflakes, or twisted boxes with large sizes (28–202 nm). The crystallite size ranging from 8 to 77 nm can be achieved at varying annealing temperatures from 300 °C to 900 °C (ref. 65) (Fig. 2).

### 2.2 Microwave-assisted method

**Fig. 3:**
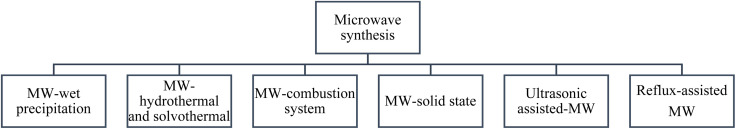
Flow sheet of the main methods in MW-assisted preparation of nano-hydroxyapatite.^69^

Microwave processing of materials is an innovative technology that provides a powerful approach for enhancing, improving, or altering the characteristics of existing materials.^66^ It is an effective way to overcome the problems associated with traditional methods. Small-sized and highly pure nanoparticles with thermal stability can be achieved through this process.^67^ The microwave synthesis method is fast and environmentally friendly. It is a time and energy-saving method, and almost 100% of the electromagnetic energy is converted into heat,^68^ resulting in uniform volumetric heating of a sample.^69^*In vitro* studies demonstrate the potential of microwave-synthesized HAp for osteoporotic bone regeneration^70^ with cell viability of more than 80%, and its bio-compatibility nature was also proven.^71^ Over the past few decades, several attempts have been made to combine microwave irradiation with other techniques^56^ (Fig. 3).

The first use of microwave (MW) irradiation to prepare pure hydroxyapatite (HAp) through precipitation from aqueous medium in under an hour was reported in 1991,^72^ where two sets of experiments were conducted in a microwave oven, one with ionic solutions to precipitate calcium phosphate species which was microwaved for 5 minutes and the other using a preformed wet solid.^73^ In later experiments, the microwave irradiation period of the reaction mixture was prolonged to 20–25 minutes.^74^ The resulting precipitate was filtered, then dried in an oven at 40 °C to 80 °C for 17–24 hours and calcined at 500–10 000 °C in most cases.^75–78^ The MW-assisted nano-HAp precipitation method successfully produced a “biomimetic” amorphous carbonate nano-HAp structure using concentrated body fluids easily and rapidly with high purity and quantity.^79^ In reflux-assisted MW synthesis, MW heating is combined with a reflux condenser to maintain the reaction temperature.^80^ With modified process parameters,^81^ this method can produce highly crystalline nano-HAp powder with smaller particle size and mixed (lenticular and rod-shaped) morphologies. The MW-hydrothermal method uses a sealed MW device at high pressure & temperature, which accelerates crystal growth and phase purity.^82^ The MW-solvothermal method is similar to the hydrothermal method, but organic solvents are used instead of water.^83^ For rapid synthesis, the MW-solid state method is preferable for being a one-step method.^84^ Experiments show that spherical nano-HAp (calcium-deficient hydroxyapatite (CDHA), 50 nm, and Ca/P ∼ 1.5) was prepared within 4 min in a domestic MW by this process.^85^ The MW-combustion system can be initiated by auto-ignition with a domestic MW oven.^86^ This method has been used for doping nano-HAp with europium for bio-imaging applications with sufficient fluorescence emission intensity.^87^ Combining ultrasonication and MW irradiation, the ultrasonic-assisted MW method enhances the surface area and mesoporosity^88^ and fastens nucleation;^89^ therefore, this process is a viable option for improving bioactivity & drug-loading efficiency. In subsequent studies, researchers have illustrated that various parameters such as aging time, microwave irradiation power, and time significantly impact HAp.^90^

### 2.3 Sol–gel method

**Fig. 4:**
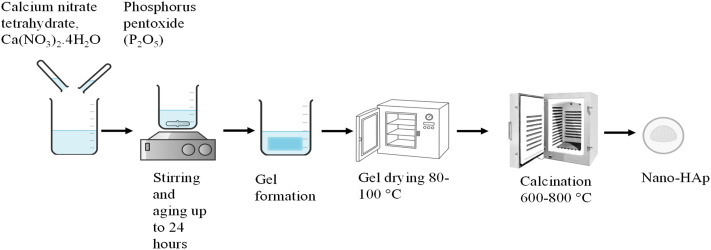
Synthesis of hydroxyapatite by the sol–gel technique.

The sol–gel method (Fig. 4) is a prominent technology in the production of nanoparticles and is widely used in industries for exceptional purity and efficiency.^91^ This method involves the transformation of a sol into a gel, followed by subsequent drying and calcination steps to obtain the desired hydroxyapatite structure. The common source of Ca is calcium nitrate tetrahydrate [Ca(NO_3_)_2_·4H_2_O] and that of P is phosphorus pentoxide (P_2_O_5_). Biocompatible sources, such as eggshell-derived calcium and trimethyl phosphate as a phosphorus source, have also been used. Water and ethanol are widely used solvents in the sol–gel method. Aging times vary from 1 hour (short process) up to 24 hours at room temperature; the drying temperature is typically kept at 80–100 °C, and calcination is done from 600 to 800 °C.^92–96^ The sol–gel process offers the advantage of creating uniform and nanostructured materials at low processing temperatures.^97^ Additionally, sol–gel coatings exhibit significant improvements in mechanical properties because of nanocrystalline grain structures.^98^ Studies indicated that the grain structure morphology in sol–gel coatings contributes to superior biological and mechanical properties.^99^ Besides, this method offers exceptional advantages, including precise control over particle size, morphology, versatility,^100^ and the attainment of high purity and homogeneity.^101^ Some of the significant drawbacks of this method are that the precursors and solvents used in this process are costly,^102^ procedures such as aging and calcination are time-consuming,^93^ and the possibility of the formation of a secondary phase of calcium oxide (CaO), which negatively impacts biocompatibility. The CaO content must be minimized through procedural adjustments or post-processing, such as washing with a dilute acid solution.^103^

### 2.4 Hydrothermal method

**Fig. 5:**
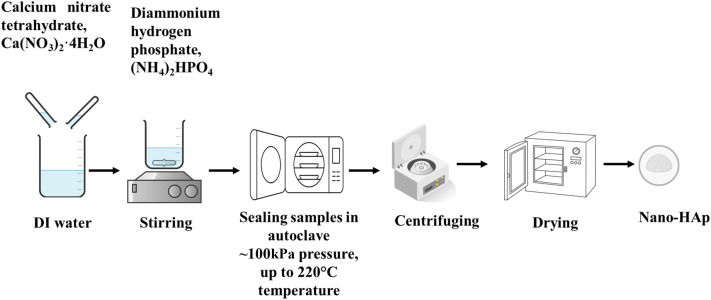
Synthesis of hydroxyapatite by the hydrothermal method.

Hydrothermal synthesis (Fig. 5) is a versatile, environmentally sustainable, and low-energy consumption method, which is used to create desired crystalline phases from slurries, solutions, or gels under mild reaction conditions.^104,105^ Nanomaterials can be synthesized across a broad temperature range^106^ with controlled size and morphology.^107^ Various compounds, including simple and complex oxides, carbonates, silicates, chalcogenides, *etc.*, are synthesized by this process. Furthermore, products with commercial value, including Be_3_Al_2_(SiO_3_)_6_ (beryl, emerald, and aquamarine), Al_2_O_3_ (corundum, ruby, and sapphire), BeAl_2_O_4_ (chrysoberyl and alexandrite), and ZnO (zincite) are grown by this method.^105^ This process typically involves dissolving calcium- and phosphate-containing substances in distilled water to make a suspension, sealing the solution in an autoclave, and treating the precipitate at controlled temperature.^108,109^ The reaction can be in a single or heterogeneous phase at pressures exceeding 100 kPa to initiate crystallization directly from solutions.^110^ The most common sources of starting material for hydrothermal synthesis of HAp include calcium nitrate tetrahydrate, Ca(NO_3_)_2_·4H_2_O, and diammonium hydrogen phosphate, (NH_4_)_2_HPO_4_. The synthesis temperature can be as low as 60 °C (minimum temperature for improved crystallinity) up to around 220 °C, the pH ranges from 3 to 11, a higher pH generally favoring the forward reaction for HAp formation, and synthesis time typically ranges from 24 to 72 hours.^111^ Maintaining such conditions for extended periods increases energy consumption, making the process costly, which is one of the drawbacks of hydrothermal processes.^112^

## 3. Importance of modifiers in nano-HAp synthesis

The characteristics and applications of nanomaterials are greatly influenced by their size, morphology, and surface chemistry. One of the most effective strategies to control these properties is through the application of modifiers during synthesis. While synthesis without modifiers can yield pure materials, they have limited control over the morphology, homogeneity, and dispersibility of nanostructures compared to modifier-assisted synthesis, which actively influences nucleation, crystal growth, and surface interactions of nanostructures.^113^ For example, in two sets of experiments on HAp synthesis by the precipitation method, one without a modifier resulted in irregular particle growth with a size range of 8.4–24 nm,^114^ while another with a modifier produced rod- and flake-shaped particles ranging from 19 to 143 nm (depending on the modifier used).^115^ This tunability is essential for optimizing nano-HAp for specific applications, such as enhanced bioactivity in bone regeneration, increased surface area for catalytic reactions, or improved dispersion in composite materials. For instance, stearic acid, as a surface modifier, prevents agglomeration, promotes better thermal stability, and improves cell viability, making the synthesized HAp highly biocompatible.^116^ Similarly, ionic surfactants CTAB or SDS were found to be highly effective in shaping the anisometric growth of nano-HAp particles, which makes them good candidates for tissue engineering applications and drug delivery systems.^117^ Exploring the specific effects of modifiers over different synthesis techniques will provide deeper insights into the importance of modifiers in nano-HAp synthesis for targeted applications.

### 3.1 Effect of modifiers on different synthesis methods

#### 3.1.1 Wet chemical technique

**Fig. 6:**
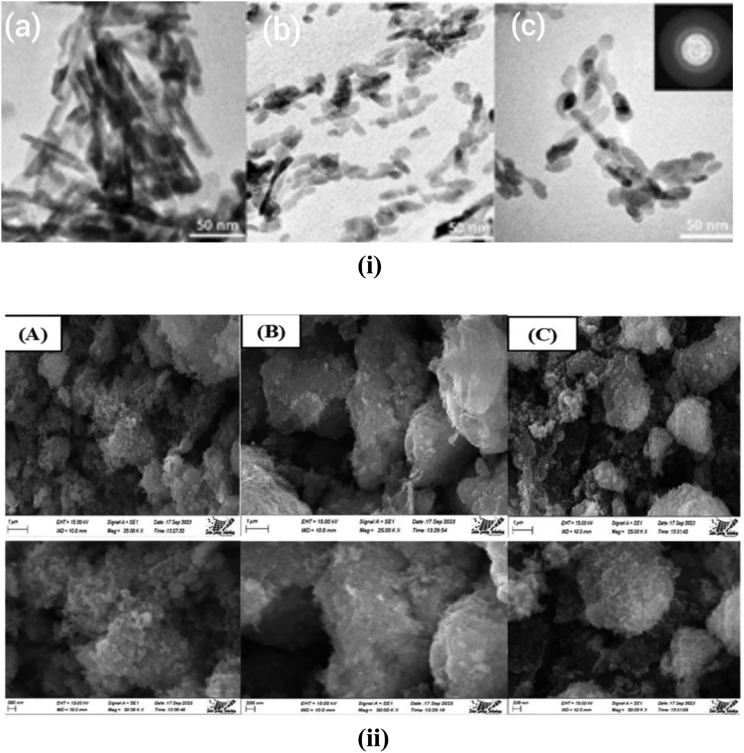
(i) TEM microphotographs of the synthesized nano-HAp: (a) HApA-10, (b) HApbPEI-10, and (c) HApLPEI-5.^124^ (ii) SEM image of HAp with different modifiers: (A) urea, (B) palmitic acid, and (C) naphthalene.^119^

HAp as a photocatalyst has been used for degrading toxic wastes and is still being explored.^118^ A study *via* the wet chemical method to enhance the photocatalytic activity of HAp indicated that modified HAp using urea, palmitic acid, and naphthalene exerted significant influence on their performance (*e.g.*, maximum degradation capacity of 7 mg g^−1^ for 100% ethanol-derived HAp). In general, these modifiers alter the crystallographic structure of HAp, creating more active surfaces for dye degradation and inducing microstrain within the crystal lattice, which affects the material's optical properties and photocatalytic efficiency. Experimental data demonstrated that urea-modified HAp showed the lowest photocatalytic activity, with only 69.63% degradation and 5.57% degradation capacity. The reason behind this poor performance could be the lowest degree of crystallinity and highest microstrain, resulting in poor photocatalytic performance.^119^ Stearic acid (SA), a surface modifier, is considered a good candidate for surface modification as it prevents particle agglomeration *via* hydrogen bond formation between the hydroxyl groups on the HAp surface and the carboxylic groups of SA. Hydrocarbon chains form a layer that stabilizes the particles, acting as a mechanical barrier limiting particle aggregation.^120^ As the experiment was conducted using two concentrations of SA (7% & 15%), the result indicated that 7% SA-coated HAp showed the best dispersion and a homogeneous structure with reduced particle size (60–77 nm), offering good bioactive composite characteristics. Excessive concentration of SA (15%) leads to larger particle aggregates due to the formation of multiple SA layers or excess SA particles forming their phase, which decreases structural integrity.^121^ Organic modifiers such as citric acid, acetic acid, glutamic acid, and gallic acid are used to control the nucleation and crystallinity of particles. In the synthesis of carbonated hydroxyapatite (CHAp), citric acid produced the smallest CHAp particles with a rod shape (19–25 nm), and since it is a strong chelator, it binds three calcium ions per citrate ion (calcium–citrate complex), mobilizing calcium ions, which influence crystal growth.^122^ Citric acid can produce smaller sized hydroxyapatite than sodium dodecyl sulphate and sodium dodecylbenzene sulphonate.^123^ Acetic acid resulted in flake-shaped particles but were larger in size as it chelated one calcium ion per acetate ion and limited calcium availability less effectively than citric acid. Glutamic acid also produced rod-shaped particles but with a slightly larger size compared to citric acid because it affected calcium availability moderately. Meanwhile, gallic acid led to the largest particles (127–143 nm) with a flake shape due to the π–π stacking interaction between CHA and GA units, promoting agglomeration. Here, temperature plays an important role; with increasing temperature, crystallinity increases.^115^ Cationic functionalized nano-HAp materials are highly promising for gene therapy. Study with the incorporation of arginine (Arg) or polyethylenimine (branched PEI – bPEI, or linear PEI – LPEI) as cationic modifiers and dispersing agents showed significant improvement in colloidal stability and DNA binding ability of HAp. Compared to Arg, the length and aspect ratio of the synthesized particle were lower in PEI with higher dispersibility due to the high content of NH_2_ free groups in PE, while LPEI was evident as most suitable to generate plate-like morphology, similar to natural bone components.^124^ As a part of an eco-friendly experiment, different concentrations (5 mg, 10 mg, and 20 mg) of caffeine, a nitrogen-containing heterocyclic compound^125^ as a modifier, were used, which improved the shape and morphology of HAp. The findings suggest that, similar to EDTA or citric acid, caffeine prevents clumping of nanoparticles^126^ and also acts as a stabilizing agent by capping the surface^127^ of forming HAp nanoparticles, leading to smaller particle size (HAp size ∼ 35 nm). Concentration should be considered before using caffeine as a modifier. A low concentration of caffeine (5 mg) did not show significant differences compared to non-modified HAp. According to TEM images, increasing concentration (20 mg) exhibited the highest crystallinity with sharp XRD peaks and well-defined rod-like morphology.^128^ Mesoporous hydroxyapatite nanoparticles were successfully synthesized using chitosan, a natural polymer. During calcination, when chitosan was separated, pores formed in the voids (average pore diameter ∼ 38 nm). Data showed that as the weight ratio of chitosan increases with varying pH, it produces larger and more interconnected pores. This tunable pore structure makes HAp highly suitable for drug delivery applications^129^ (Fig. 6).

An overview of the response to using various modifiers in the synthesis of nano-HAp by the wet chemical method is provided in Table 2. This table highlights the key characteristics and outcomes associated with each modifier, offering a clear comparison of their effects.

**Table 2:** An overview of the effects of modifiers in synthesizing nano-HAp by the wet chemical method^a^

Starting materials	Temp. (°C)	pH	Organic modifiers	Morphology	Particle size (nm)	Crystal size (nm)	Crystallinity	Ca/P	Pore size (nm)	Surface area (m^2^ g^−1^)	Application	Ref.
CaCl_2_·2H_2_O and 85% H_3_PO_4_	Room temp. (RT) to 80 °C	9	Citric acid	Rod-shaped	L: 19–25	—	28.6%	1.88	—	—	Dentistry, drug delivery system, and bone tissue engineering	115
					W: 13–20							
			Acetic acid	Flake-shaped	L: 55–68		30%	1.66				
					W: 22–50							
			Glutamic acid	Rod-shaped	L: 33–43		27.7%	1.68				
					W: 16–31							
			Gallic acid	Flake-shaped	L: 127–143		25%	1.71				
					W: 56–122							
Ca(OH)_2_ and H_3_PO_4_	—	10–11	Naphthalene	Spherical	—	9.06	0.012	1.67	—	0.20	Photocatalysis	119
			Palmitic acid	Spherical		11.59	0.020			0.163		
			Urea	Agglomeration		11.50	0.0038			0.165 (theoretical calculation)		
Ca(NO_3_)_2_·4H_2_O and NH_4_NaHPO_4_·4H_2_O	RT	9	Arginine	Needle-like	L: 71.3	—	92.1%	1.56	—	—	Gene therapy	124
					W: 7.9							
			Polyethylenimine branched (bPEI)	Plate-like	L: 45.9		92.2%	1.63				
					W: 9.4							
			Linear polyethylenimine (LPEI)		L: 32.2		94.6%	1.61				
					W: 17.4							
Ca(NO_3_)·2H_2_O and (NH_4_)_2_HPO_4_	RT	12	Caffeine, 5 mg	Nanorods clear in shape and size with increasing caffeine concentration	25–35	28	Improves with concentration	—	—	Larger	—	128
			10 mg			32						
			20 mg			35						
Ca(NO_3_)_2_·4H_2_O and NaH_2_PO_4_		8	Chitosan, 0 g	Spherical	—	38		1.36	28.5	21.3	Slow-release drug delivery and osteoporosis treatment	129
		9	0.1 g			34		1.48	29	36.5		
		10	0.3 g			24		1.49	38.6	41.7		
Calcium nitrate tetrahydrate and phosphoric acid (85%)	40 (post-treatment at 100 and 200 °C)	10	Citric acid	Rod-like	(At 100 °C) L: 15	—	Increases with increasing synthetic temp.	1.67	—	—	—	123
					W: 9							
			Sodium dodecyl sulphate		L: 21			1.67				
					W: 11							
			Sodium dodecylbenzene sulphonate		L: 25			1.66				
					W: 12							
Calcium nitrate tetrahydrate and phosphoric acid (85%)	40 (post-treatment at 100 and 200 °C)	10	Polyethylene glycol (MW: 600)	Nanorod	(At 100 °C) L: 13	—	—	1.66	—	—	—	130
					W: 30							
			Tween 20	Dendriform	L: 12			1.67				
					W: 23							
			Trisodium citrate	Nanorod	L: 10			1.65				
					W: 17							
			d-Sorbitol	Linear	L: 12			1.66				
					W: 25							
Ca(OH)_2_ and H_3_PO_4_	25	5	Lactic acid	Plate-like	∼19	—	—	1.05	—	—	Biomedical	131
		7		Spherical	∼111			1.32				
		10			∼86			1.44				
		12			∼48			1.55				
Ca(NO_3_)_2_·4H_2_O and NH_4_HPO_4_	RT	3	PEG 600	Small agglomerates	50–60	—	—	—	0.18 cm^3^ g^−1^	23	Wastewater purification	132

a All surface area and pore size measurements in this review paper were performed using the Brunauer–Emmett–Teller (BET) and the Barrett–Joyner–Halenda (BJH) methods, respectively, unless otherwise stated.

#### 3.1.2 Microwave-assisted method

**Fig. 7:**
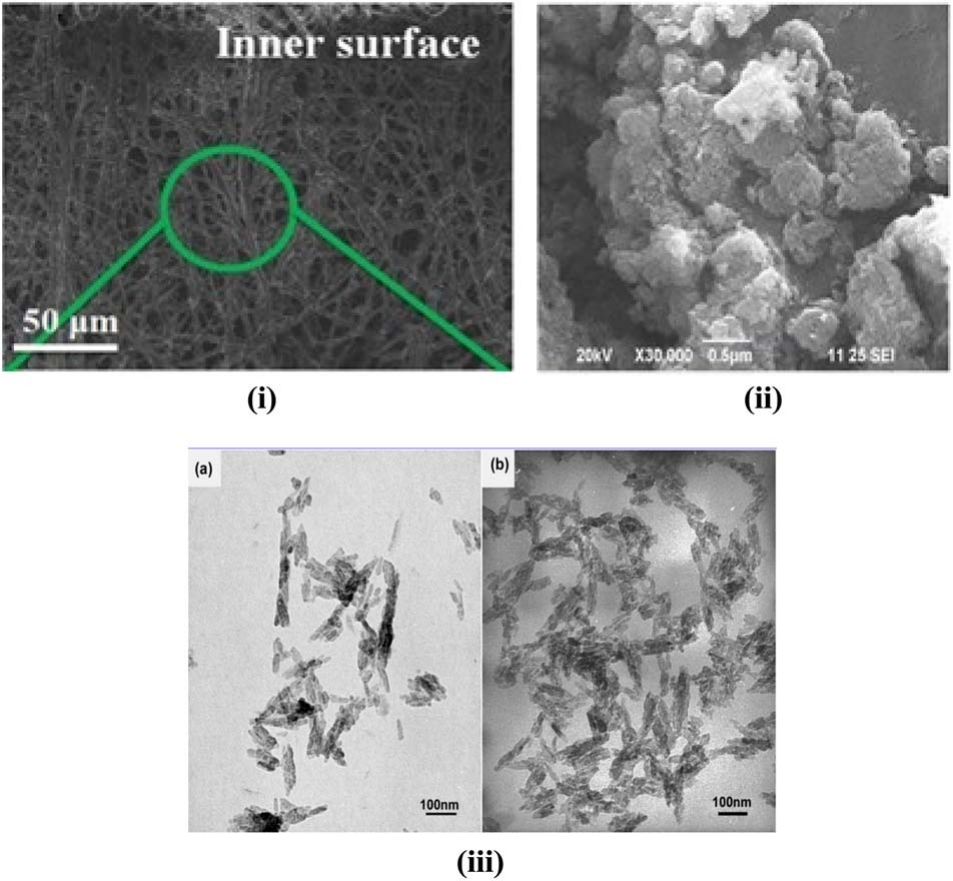
(i) SEM image of citric-acid assisted HAp,^158^ (ii) SEM image of *Moringa oleifera* flower extract capped hydroxyapatite,^147^ and (iii) TEM images of HA powders prepared in the presence of (a) 15% alginic acid and (b) 15% sodium alginate.^150^

Organic modifiers such as EDTA, amino acids, CTAB, polyvinylpyrrolidone (PVP), and trisodium citrate showed significant influence on the morphology, crystallinity, and biocompatibility of HAp.^133^ EDTA is a water-soluble polymer commonly used as a chelating agent and also as a complexing agent. It has been used as a capping agent for preparing various metal nanoparticles, including gold (Au), zinc (Zn), copper (Cu), and chromium (Cr).^134,135^ Capping agents have clinical significance for modifying nanoparticles that are biocompatible^136^ as surface capping enhances the biological properties and modifies the properties of colloidal suspensions.^134^ EDTA^4−^, a complex reagent, is a member of the poly-amino carboxylic acid family. It acts as a hexadentate ligand while binding with Ca^2+^ ions, surrounding each Ca^2+^ ion with four oxygen atoms and two nitrogen atoms and forming several chelate rings in a stable Ca–EDTA complex.^137^ Stable Ca–EDTA complexes control the crystal by modulating the availability of Ca^2+^.^138^ One study using EDTA as a capping agent in the synthesis of hydroxyapatite (HAp) at varying pH^9,11,13^ showed spectroscopic characteristics along with structural characteristics. The IR spectra of the samples indicated that EDTA-assisted samples are purer and uniform. The EDTA-assisted samples were structurally well-defined with smaller particle sizes (∼100 nm) and reduced carbonate contamination compared to the samples without EDTA.^139^ Temperature plays a crucial role here with a higher sintering temperature of 1100 °C (pH 9), facilitating anisotropic growth, forming larger nanostrips.^138^ Also, data indicated that at higher pH, the samples are more uniform and dispersed.^139^ Similar to EDTA, oxalic acid, a chelating agent, forms calcium oxalate driven by the strong electrostatic attraction between the oxalate anions (C_2_O_4_^2−^) and calcium cations (Ca^2+^), which allows for controlled release of Ca^2+^ ions and prevents premature crystallization of HAp. In addition, oxalic acid increases the surface area and produces mesoporous HAp, which is highly preferable for drug delivery applications.^140,141^ Cationic–anionic surfactants possess better adsorbent properties and are ideal for adsorption of dyes and metal ions. A study showed that the use of cationic–anionic surfactants (CTAB, sodium dodecylbenzene sulfonate (SDBS), and SDS) caused the surface area of HAp nanorods to increase (the surface area for individual anionic counterpart – 52 m^2^ g^−1^; for cationic – 48 m^2^ g^−1^; without surfactant – 19 m^2^ g^−1^; with a mixture of cationic–anionic surfactants, it was higher – 56 m^2^ g^−1^). They evaluated the adsorption capacity and found the maximum amount of dye adsorbed (methylene blue) was 833 mg g^−1^.^137^ CTAB has a potential ability to facilitate micelle formation. As the concentration increases, it reacts progressively to the PO_4_^3−^ groups and creates an electrostatic barrier, effectively inhibiting longitudinal growth and yielding nanorods with smaller dimensions.^142,143^ However, at much higher concentrations above the critical micelle concentration (CMC), flexible worm-like micelles form, providing elongated templates for particle growth.^144^ Chitosan can generate well-dispersed nanoscale HA particles embedded in a polymeric matrix with a uniformly porous interconnected network. It is non-toxic to MG 63 osteoblasts with cell viability of up to 54.5%.^145^ The modifiers typically used have exhibited some degree of toxicity, so researchers are shifting towards bio-friendly growth regulators, particularly those that naturally occur in the body, such as amino acids (glycine, serine, etc.). The electrostatic interaction between amino acids and the outer surface of nanocrystals of HAp leads to morphological changes. Adsorption of amino acids can occur on any specific crystallographic face, inhibiting growth in the perpendicular direction while allowing growth parallel to the face, which results in a larger surface area. They significantly reduce hydroxyapatite's aspect ratio and crystallinity, increasing the cytocompatibility.^146^*Moringa oleifera* flower extract is another biofriendly option prepared by boiling dried moringa flowers and is rich in tannins and polyphenols. They act as chelating agents and enhance the structural and biological performance of synthesized HAp^147^ (Fig. 7 and Table 3).

**Table 3:** A summary of the effects of different types of modifiers in the structural variation of nano-hydroxyapatite synthesized by the microwave method

Starting material	MW power (W)	MW time (min)	Sintering temp. (°C)	pH	Modifier	Morphology	Particle size (nm)	Crystal size (nm)	Surface area (m^2^ g^−1^)	Pore size (nm)	Yield (%)	Ca/P	Application	Ref.
Ca(NO_3_)_2_·4H_2_O and (NH_4_)_2_HPO_4_	800	45	1100	9	EDTA	Uniform nanostrips	L: 50–100	30–50	—	—	—	—	—	138
Calcium nitrate tetrahydrate and diammonium hydrogen phosphate	750	30	900	9	EDTA	Capsule-like	D: ∼10	—	—	—	—	—	Biomedical	139
				11		Scattered needle-like	L: ∼100							
				13		Flower-like								
CaNO_3_·4H_2_O and Na_2_HPO_4_	700	30	—	11	EDTA	Bow knot-like	W: 150	—	—	—	—	—	—	148
				13		Flower-like flakes	W: 150–200							
Ca(NO_3_)_2_·4H_2_O and KH_2_PO_4_	700	20	—	13	Oxalic acid	Loosely agglomerated nanorods	L: 20–40	—	89	—	—	1.77	Biomedical	140
					SDS		L: 45–100		48			1.55		
Ca(NO_3_)_2_·4H_2_O and NH_4_H_2_PO_4_	—	10	550	10	CTAB	Rod-shaped	L: varies from 80–120	—	48.8	—	—	—	Dye and heavy metal absorption	149
					SDS				52.8					
					SDBS				50.4 (BET)					
Ca(OH)_2_ and (NH_4_)_2_HPO_4_	800	300	—	11	Sodium alginate 5%	Aggregated nanocrystals	L: 50	25	—	—	—	—	Biomedical	150
					10%			24						
					15%			22						
					Alginic acid 5%			24						
					10%			22						
					19%			19						
Ca(NO_3_)_2_·4H_2_O and (NH_4_)_2_HPO_4_	900	20	—	—	EDTA	Spherical	22	—	—	—	—	1.686	Electrochemical sensing of uric acid	151
Ca(NO_3_)_2_·4H_2_O and (NH_4_)_2_HPO_4_	300	20 s	—	—	Chitosan	Porous	50–70	65	—	112–343 μm (scaffold)	—	1.65	Bone tissue engineering	145
Ca(OH)_2_ and H_2_PO_4_	35	10	100	12	*Moringa oleifera* flower extract	Rod-like	41	18.6	—	—	—	1.81	Antimicrobial	147
Eggshells and NH_2_HPO_4_	900	—	900		EDTA	Flower-like	500 nm to 1.5 μm	—	—	—	At pH 13 19.6	1.37	Biomedical	152
				11	0.1									
				12	0.2						50.4	1.51		
				13	0.3						84.3	1.62		
Ca(NO_3_)_2_·4H_2_O and (NH_4_)_2_HPO_4_	—	30	500	>10	Sodium lauryl ether sulfate (SLES)	Rod-like	L: 52	19.9	48	35.4	—	1.67	—	153
							W: 18							
					Linear alkylbenzene sulfonate (LABS)		L: 80	19.2	60 (BET)	35.3 (BJH)				
							W: 20							
Eggshells and NH_2_HPO_4_	600	10 min	—	8	EDTA	Flower-like	L: 0.5–1 μm	—	—	—	—	1.65	Drug delivery	154
							W: 100–200 nm							
Ca(NO_3_)_2_·4H_2_O and (NH_4_)_2_HPO_4_	400	45	—	6.1	Licorice root extract	Rod-like	L: 105	38	—	—	—	1.69	Biomedical	155
							W: 25							
Ca(NO_3_)_2_·4H_2_O and (NH_4_)_2_HPO_4_	800	30	—	10	EDTA	Mixed	L: ∼71	23–34	20.63	2.29	—	—	—	156
					CTAB	Mixed	W: ∼16	26–33	22.65 (BET)	4.3				
Black *Chlamys varia* seashell and K_2_HPO_4_	700	20	—	13	SDS	Rod-like	L: 300–600	—	49	2.46	—	1.42	Biomedical	157
							W: 10–15							
Eggshells and potassium phosphate	700	30	—	13	Citric acid	Rod-like	L: 7–10	—	58.3 (BET)	7	—	1.86	Biocidal implant application	158
							W: 20–30							
Ca(NO_3_)·4H_2_O and (NH_4_)_2_HPO_4_	—	30	—	10	CTAB	Rod-like	L: 242–136	51–47	—	—		1.69	Gas sensing and biomaterial applications	159
Ca(NO_3_)·4H_2_O and (NH_4_)_2_HPO_4_	—	30	—	9	(Amino acids) glycine	Irregular changes with concentration	Length 100–53	—	70–93	—	—	—	Scaffolds and drug delivery	146
					Serine		85–55		78–86					

#### 3.1.3 Sol–gel method

**Fig. 8:**
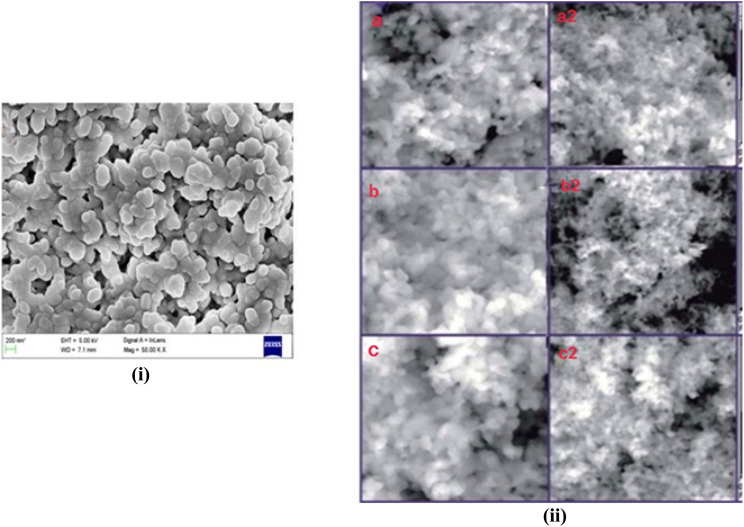
SEM images of (i) modifier (0.1 M CTAB)-assisted HAp spheroidal particles^163^ and (ii) stearic acid-assisted HAp at pH 7 (a and a2), pH 9 (b and b2), and pH 11 (c and c2), with uniform rod-like structures at 40k and 80k times magnification of the sample.^170^

For targeted applications, researchers started applying modifiers in the sol–gel method to fine-tune the characteristics of nano-hydroxyapatite. Organic modifiers such as trisodium citrate, citric acid, polyethylene glycol, Tween 20, d-sorbitol, ethylene glycol, and sodium dodecyl sulphate are widely used.^160^ Mesoporous hydroxyapatite (MPHA) is highly biocompatible with a high surface–volume ratio and adsorption capability. To synthesize MPHA, stearic acid (SA), a surface modifier,^161^ resulted in high surface area, porosity, and pore size (5.84 nm – BET analysis), and excellent cytocompatibility with high cell viability (up to 83%). It was suggested that the carboxyl group in stearic acid likely adhered to the surface of the HAp during the process, creating small, uniformly distributed pores. Also, the strong interaction between SA and ethanol organized cylindrical structures (micelles), helping to create rod-like HAp. pH was a pivotal parameter here. Only pH 11 resulted in a well-defined structure, while pH 7 and 9 contained impurities like β-TCP (FESEM analysis) with a sponge-like structure.^162^ As a templating agent, CTAB can also produce porous HAp ranging from 6 to 10 nm with varying concentrations of CTAB.^163^ Another templating agent, polyethylene glycol (MW 600), modifies the morphology of particles where nano-HAp appears to be agglomerated with sub-microscopic pores. Its flexibility allows its chains to interact with hydroxyapatite nanocrystals. The ether bonds (–O–) of polyethylene glycol interact with HAp nanocrystals, guiding them to grow in a specific direction. The flexibility is highly temperature sensitive in aqueous solution, which is a drawback of polyethylene glycol. At higher temperatures, it acts like a soft template, encouraging organized, oriented growth along certain axes and promoting the formation of flat, platelet-like HAp structures instead of random shapes, which is beneficial for biomedical use. A study using sintering temperatures of 400 °C, 750 °C, and 1100 °C showed that only at 1100 °C were the X-ray patterns aligned with ASTM data.^164^ Citric acid is quite a common modifier used in the synthesis process of HAp.^165^ When citric acid is used as a modifier, it forms a calcium–citrate complex. This chelating effect moderates the availability of free calcium ions in solution and limits the size of the HAp particles formed.^166^ While synthesizing hydroxyapatite–calcite, data indicated that at 60 °C samples with citric acid modifiers produced smaller sized particles (55 nm) with lower crystallinity compared to samples without citric acid (particle size ∼ 84 nm), which is a good fit for biomedical applications. Temperature, pH, and concentration of citric acid are vital in these preparations (*e.g.*, at room temperature, the particle size reduces to 51 nm from 55 nm (at 60 °C)).^160^ Lemon extract, a natural source of citric acid, has also been used, and the resultant HAp showed well-defined characteristics with suitability for cancer treatment.^167^ EDTA as a chelator can form mesoporous HAp with a surface area of up to 155 m^2^ g^−1^ with robust ion exchange capacity, making it an efficient absorbent for radioactive materials (55–63% uptake of U(vi) and Cs(i) within 10 minutes) with maximum adsorption capacities of 77.2 mg g^−1^ for Cs(i) and 59.3 mg g^−1^ for U(vi).^168^ Latex and carbon fibers work as pore-forming templates and can generate HAp with micro-, meso-, and macropores (up to 100 nm) with a high surface area^169^ (Fig. 8 and Table 4).

**Table 4:** An overview of the effects of modifiers in synthesizing nano-HAp by the sol–gel method

Starting material	Solvent	pH	Reaction temp. (°C)	Modifiers	Morphology	Ca/P	Particle size (nm)	Crystal size	Crystallinity	Specific surface area (m^2^ g^−1^)	Pore size (nm)	Application	Ref.
Ca(NO_3_)_2_·4H_2_O, 98%, (NH_4_)_2_HPO_4_, 99%	Deionized (DI) water	>10	Room temp.	Citric acid	Agglomerated (calcined at 400 °C)	—	51	—	3.61	—	—	Biomedical	160
			60				55		4.35				
Ca(NO_3_)_2_·4H_2_O and (NH_4_)_2_HPO_4_	Ethanol	10	85	5% polyethylene glycol	Agglomerated (with sub-micrometric pores)	—	50–70	—	—	—	Sub-micrometric	Biomaterial in bone implantation	164
Ca(NO_3_)·4H_2_O and (NH_4_)_2_HPO_4_	Ethanol	7	—	Stearic acid	Rod-like (uniform)	1.602	—	11	(%) 1.749	∼7.7138	5.85	Drug delivery and bone tissue engineering	170
		9				1.55		11	1.757	∼10.519			
		11				1.68		10	1.799	∼66.265			
Ca(NO_3_)_2_·4H_2_O	Diluted water and ethanol	—	48	Polyethylene glycol	Needle-shaped	—	—	Avg 40–50	—	—	—	—	171
				Acetic acid	More agglomerated								
				Together	Complex morphology								
Ca(NO_3_)_2_·4H_2_O and H_2_PO_4_	Distilled water	9	50–120	Lemon extract	Spherical	1.59	25–35	—	—	—	—	Cancer treatment	167
Ca(NO_3_)_2_·4H_2_O and (NH_4_)_2_HPO_4_	Double distilled Millipore water	11	70	CTAB (0.1 M)	Spheroid	—	20–100	—	—	51.8	7.83	Drug delivery, antioxidant	163
CaCO_3_ and (NH_4_)_2_HPO_4_	Distilled water	11	80	Alginic acid	Agglomerated	—	50–100	—	—	—	—	Biomedical application	172
Ca(NO_3_)_2_·4H_2_O and H_2_PO_4_	Ethanol/DIW	7.9	70	EDTA	Irregular flaky-flower	—	—	—	—	155	4.2	Adsorbent for radioactive ion remediation	168
Ca(NO_3_)_2_·4H_2_O and P_2_O_5_	Alcoholic and aqueous solutions	3–9	60	CTAB	Spherical	—	50	—	—	—	2 density functional theory (DFT)	Coating material	173
CaCl_2_·2H_2_O, (NH_4_)_2_HPO_4_	Water		90	Siloxane-acrylate latex and carbon fibers	Needle-like	—	—	—	—	61.7	50–100	Bone tissue regeneration	169
Ca(NO_3_)_2_·4H_2_O and (NH_4_)_2_HPO_4_	Deionized water	—	150	Natural rubber latex	Plate-like	1.67	30–72	65–74 (calcination temp.: 600)	1.6–2.3	—	—	—	174

#### 3.1.4 Hydrothermal method

**Fig. 9:**
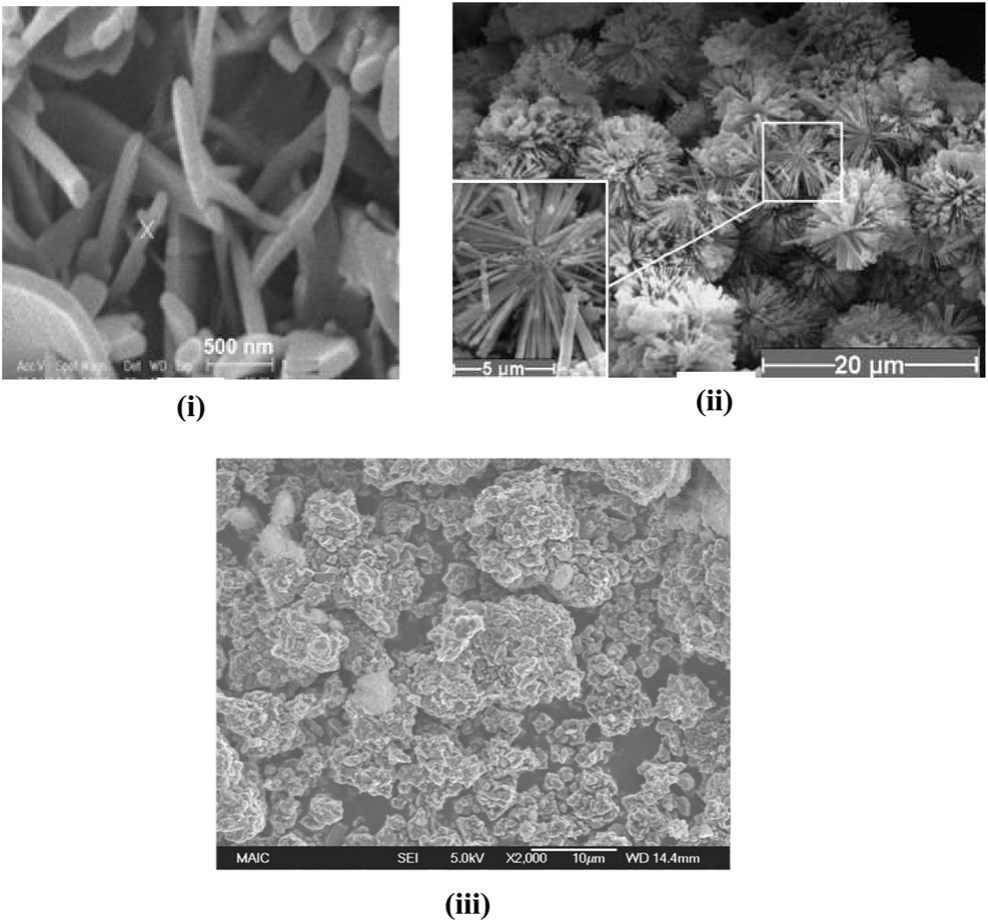
SEM images of HAp synthesized with different modifiers: (i) PEG 400 assisted rod-like HAp,^194^ (ii) EDTA assisted dandelion-like structure,^185^ and (iii) aminotris assisted spherical nanoparticles of HAp.^188^

Amino acids such as glutamine, alanine, and valine can tailor the morphology and crystallinity of HAp for efficient application.^175^ A study using glutamine in the hydrothermal synthesis, which is simple and cost-effective, produced nano-rods of HAp with average lengths ranging from 50 to 100 nm. Being a biomolecule, glutamine is non-toxic and environmentally friendly. They can imitate biomineralization, a natural process (biomimetic method).^176^ Another eco-friendly option is alginate, a naturally occurring linear polysaccharide commonly found in different brown seaweeds. As a modifier, they adsorb onto the surface of specific crystallographic planes of HAp nuclei, which blocks further ion attachment along the *c*-axis. Under hydrothermal conditions, alginate depolymerizes to oligosaccharides and monosaccharides. These anionic groups actively adsorb onto the surface.^177^ In an experiment with three different concentrations of alginate (HA-0.4%, HA-0.8%, and HA-1.6%) and a sample without alginate, the effect of alginate on the morphology of HAp was evident. The XRD analysis revealed that with increasing concentration of alginate, the crystallinity decreases, and the particles become smaller and more aggregated. The SEM images showed that nanoparticles become more dispersed at higher alginate concentrations. If the goal is to get a well-defined rod-like structure, then glutamine is more suitable, as alginate gives less defined rod-like structures of HAp.^178^ Saponin, a plant-derived surfactant, forms micelle-like structures that influence crystal nucleation and growth of the synthesized HAp. With increasing concentration of saponin, the nanorods become thinner and more acicular. It contributes antifungal and antibacterial properties and enhances the surface activity of HAp.^179^ For mesoporous HAp under hydrothermal conditions, the use of Triton X-100 resulted in increased pore volume. Triton X-100 is a non-ionic surfactant that hinders clumping of HAp particles through van der Waals interactions, contributes to particle stability in suspension, leads to the formation of rod-like structures following oriented attachment of crystal growth (at high concentrations), and under hydrothermal conditions alters the pore structure (increases the pore volume). Although Triton X-100 has a similar hydrophilic side chain as polyethylene glycol (PEG), both show differences, while even at higher concentrations Triton X-100 does not have any impact on the crystallization process,^180^ and PEG, on the other hand, influences crystal growth, which is mediated by temperature. With increasing temperature, crystallinity increases.^181,182^ Different approaches of the hydrothermal method, including the use of novel techniques such as sono-chemical for biomedical applications along with organic surfactants like CTAB, can control the properties of particles. SEM analysis shows a well-defined rod-like structure with a diameter of 30–50 nm, which remained intact even after prolonged ultrasonic treatment. Residence time in the autoclave is an important parameter here; TEM images revealed that with increasing time (20 h), the diameter changes to 15–40 nm.^183^ Furthermore, CTAB showed contradictory outcomes in the present study, where the former study encouraged that CTAB likely favors the growth of HAp crystals along the *c*-axis,^51^ and the study suggested that CTAB can block the growth along the *c*-axis. CTAB and PO_4_^3−^ both have tetrahedral structures; this structural complementarity and the electrostatic effect sometimes lead to the adsorption of CTAB ions on the (001) planes of HAp, which may block the crystal growth along the *c*-axis, producing shorter nanorods rather than longer ones.^184^ SDS is another surfactant that can be used instead of CTAB for its similar effect.^183^ The EDTA-assisted hydrothermal process was used to synthesize a complex three-dimensional dandelion-like HAp, as EDTA controls agglomeration, growth, and is also cost-effective. Dandelion-like HAp possesses a high specific surface area and relevant properties for catalysts and molecular sieves.^185^ The mechanism here can be described as involving the Ca–EDTA complex (mentioned in 2.2.1), which facilitates the radial self-assembly into dandelion-like HAp^186^ (Fig. 9 and Table 5).

**Table 5:** An overview of the effects of modifiers in synthesizing nano-HAp by the hydrothermal method

Starting materials	Modifiers	Temp. (°C)	Time (hour)	pH	Morphology	Particle size (nm)	Crystallinity	Pore size (nm)	Pore volume (cm^3^ g^−1^)	Ca/P	Application	Ref.
Ca(NO_3_)_2_·4H_2_O and (NH_4_)_2_HPO_4_	Triton X-100 (4% or 8%)	100	6	10.4	Nano-rods	31–90 (4%)	—	7.4–55	—	—	Catalysis	180
						33–159 (8%)						
Ca(OH)_2_ and H_3_PO_4_	Urea	180	3	10–11	Small aggregates	38.745	0.154	—	—	1.67	Drug delivery	186
	Naphthalene				Controlled growth	53.236	0.308					
	Palmitic acid				Excessive aggregated	50.798	0.027					
Ca(NO_3_)_2_·4H_2_O and H_3_PO_4_	Trisodium citrate	100	8	10	Nano-rods	17.6	Increases with autoclaving temp	—	—	1.61	—	187
	Tween 20					23.0				1.65		
	Polyethylene glycol (MW: 600)					30.2				1.66		
CaCl_2_ and NaH_2_PO_4_·2H_2_O	Alginate 0.4%	200	5	12	Less-defined rod-like particles	44	Decreases with concentration		0.380 cm^3^ g^−1^	—	Tissue engineering	178
	0.8%					38			0.327 cm^3^ g^−1^			
	1.6%					33			0.335 cm^3^ g^−1^			
CaHPO_4_·2H_2_O	CTAB	150	2	—	Rod-like	50–30	Increases with concentration	—	—	1.67	Clinical application	183
Ca(NO_3_)_2_ and Na_3_PO_4_	CTAB and SDS	150	10	—	Nano rods	150	—	—	—	1.59	—	51
	PVA				Aggregates							
CaCl_2_ and K_2_HPO_4_	EDTA	50	1.5	12	Dandelion-like	200 avg (individual nanorods)	Increases	—	—	—	Catalyst, molecular sieves, and biosensors	185
Ca(C_2_H_3_O_2_)_2_·H_2_O and H_3_PO_4_	Aminotris (methylene phosphonic acid) (ATMP)	140	6–24	9	Spherical shape	30–80	—	—	—	—	—	188
CaCl_2_ and H_3_PO_4_	CTAB	90	24	10–10.5	Rod-like	L: ∼75	—	—	—	—	Biomedical	189
	SDS				Thin rods	L: ∼137						
	Triton X-100				Rods & spheres	L: ∼79						
Hydroxyapatite	EDTA	70	6	6	Agglomerated rods	>100	—	—	—	—	Filler for dental restorative materials	190
	SDS			8	Smallest rods							
	Apricot tree gum (ATG)			10	Largest rods							
Ca(NO_3_)_2_·4H_2_O and (NH_4_)_2_HPO_4_	Polyvinylpyrrolidone (PVP)	120–180	12	—	Compact spheres (180 °C)	10 μm	—	—	—	—	Biomedical	191
Ca(NO_3_)_2_ and (NH_4_)_2_HPO_4_	Fruit extract mango	180	24	10–11	Rod-like	L: 265	60%		—	—	Manufacture of bone substitutes	192
	Grape					L: 148	65%					
	Tamarind					L: 222	55%					
Ca(NO_3_)_2_ and (NH_4_)_2_HPO_4_	*Ceiba pentandra* (KAPOK)	180	20	10	Tubular	10.32–45.5 (inner diameter)	—	—	—	—	Drug delivery	193
Ca(NO_3_)_2_·4H_2_O and (NH_4_)_2_HPO_4_	CTAB	90–150	22	7	Rod-like	50–80 (150 °C)	—	—	—	—	Biomaterial	194
	PEG 400											
Ca(NO_3_)_2_·4H_2_O and (NH_4_)_2_HPO_4_	Saponin (0.5 to 5 g)	200	5	11	Rod like (0.5 g)	L: 72–150 (0.5 g)	2.23 (0.5 g)	—	—	1.59	Bone regeneration and antimicrobial coatings	179
					Needle like (5 g)		3.31 (5 g)					
CaCl_2_ and K_2_HPO_4_·3H_2_O	CTAB	40	24	12	Ill-defined clusters	—	—	5.2	—	—	Biomedical	195
		80			Needle-like			2.1				
		160			Rod-like			1.9 (not mentioned)				
CaCl_2_ and K_2_HPO_4_·3H_2_O	CTAB	60–150	2	12	Rod-like	D: ∼20–15	—	—	—	—	—	196
						L: ∼60–150 (varies with temperature and time)				—		
Ca(OH)_2_ and H_3_PO_4_	Succinic acid	180	3	10–11	Cluster-like	—	(16.1 ± 0.5) × 10^−3^	—	—	—	Bone implants and drug delivery systems	249
	Ascorbic acid				Cloud-like	—	1.49 ± 0.05	—	—	—	Biomedical uses	
	Stearic acid				Rod-like or needle-like	—	4.096 ± 0.1	—	—	—	Energy storage materials	
CaCl_2_·2H_2_O and K_2_HPO_4_	EDTA	120	4	5	Plate-like	—	—	—	—	1.41	—	197
		140		7	Thin blade	5–10 μm				1.59		
				9	Thin blade	1.5 μm in length				1.61		

#### 3.1.5 Special class of modification

Doping hydroxyapatite (HAp) with foreign ions has gained much recognition in recent times for effectively altering properties such as size, morphology, surface charge, porosity, and topology, compared to other structural modifications.^198^ Different dopants such as Zn, Cu,^199^ Mg,^200^ Sr,^201^ Ag,^202^ Mn,^203^ Se, and F^204^ are used for biomedical applications, coating materials,^205^ anti-microbial effect,^206^ human hepatoma cells,^207^ catalytic activities,^208^ and so on. The choice of dopant depends on its functionality, structural compatibility, solubility, mechanical enhancement, and application. More than half of the elements in the periodic table can be incorporated into the HAp structure. Considering the potential toxicity and radioactivity of certain elements, some have not been tested yet. 72 out of 118 elements have been successfully incorporated into HAp, representing 61% of the periodic table.^209^ They can be introduced as single elements or binary and multiple.^210^ Single incorporation is done for altering specific properties, such as Mg to improve cellular behavior.^211^ Binary or multi-ions enhance multi-functionalities, for example, Sr–Zn doping maintains a higher HAp phase percentage (>93%) and crystallinity higher than the value of 71%,^212^ and Mg, Si, and CO_3_ together enhanced the solubility rate of HAp with ion release for a longer period.^213^ The synthesis process of single ion doping in the HAp structure is easier and less complex than that using binary or multiple ions (Table 6).

**Table 6:** An overview of the effects of dopants on nano-HAp

Starting material	Method	Dopant	Additive	pH	Reaction temperature (°C)	Morphology	Particle size (nm)	Crystallinity	Crystal size (nm)	Surface area (m^2^ g^−1^)	Application	Ref.
Ca(NO_3_)_2_·4H_2_O and (NH_4_)_2_HPO_4_	Chemical co-precipitation	Mg	—	11	80	Spherical nanoparticles with a uniform distribution	∼93.3	Reduced	—	—	Antimicrobial activity	214
CaCl_2_·2H_2_O and H_3_PO_4_	Modified sol–gel technique	Europium (Eu)	Triethylamine (TEA) and dimethyl sulfoxide (DMSO)	10	Room temp.	Short rod-shaped	∼48 (length)	—	4.29 (1% Eu)	—	Bioimaging	215
		Samarium (Sm)				Mixed	∼74 for long, ∼28 for short		4.78 (1% Sm)			
Ca(NO_3_)_2_·4H_2_O and (NH_4_)_2_HPO_4_	Reflux method	Fe 0.05 M	—	10	70	Spherical-shaped with slight agglomeration (0.05 and 0.1 M) and needle-like (0.2 M)	—	Decreases with increasing Fe concentration	6.63	—	Anti-bacterial	216
		0.1 M							4.5			
		0.2 M							3.53			
Ca(NO_3_)_2_·4H_2_O and (NH_4_)_2_HPO_4_	Sol–gel synthesis	Ce	—	—	40	Ellipsoidal	∼10 to 25 nm	—	9.4 ± 0.5 nm	—	Antifungal	217
CaCl_2_·2H_2_O and (NH_4_)_2_HPO_4_	Co-precipitation	Zn	Casein	—	30	Rod-like	27 (1%)	Reduced	—	138–182	Antimicrobial	218
							26 (2%)					
Ca(OH)_2_ and H_3_PO_4_	Wet-chemical	Cu	—	10–11	25	Rod-like	—	∼2–6.7	∼65–43	—	Dye degradation	219
Ca(NO_3_)_2_ and (NH_4_)_2_HPO_4_	Wet chemical	Pd	—	9.4–9.5	Room temp.	Highly agglomerated	58.2	—	Larger crystal	—	Electrocatalytic detection of hydrazine	220
Ca(NO_3_)_2_ and (NH_4_)_2_HPO_4_	Combustion method	Ag–F	Urea	—	600	Rod-like	8 to 63	—	—	—	Biomedical	221
Ca(OH)_2_ and H_3_PO_4_	Wet precipitation	Zn (5%)	—	11	Room temp.	Needle-like	10–20	0.330	23	—	Drug delivery	222
Ca(OH)_2_ and H_3_PO_4_	Chemical precipitation	Ag	Trisodium citrate and urea	12.5	100	—	58	87%	—	—	Orthopedic and body implantation	223
HAp	Ion-exchanged method combined with calcination	Co	—	—	Ambient temp	—	150	—	—	45–52	Catalyst	224
Calcium nitrate tetrahydrate and potassium dihydrogen phosphate	Surfactant-mediated approach	Co	Triton X-100 and nitric acid	—	—	Needle-shaped	30–60	—	52–16	—	Implant and reconstructive surgery applications	225
Ca(NO_3_)_2_·4H_2_O and (NH_4_)_2_HPO_4_	Sol–gel	Ni	—	10	—	Spherical	40–50	—	39.91	—	Orthopedic and surgical procedures	226
Ca(NO_3_)_2_·4H_2_O and (NH_4_)_2_HPO_4_	Wet-chemical	Ni	CTAB	10–12	80	Spheroid shape	15–25	22–42%	111.7–83	—	Bone tissue engineering	227
Cow's cortical bone	Ball-milling	Li	Stearic acid	—	—	Cauliflower-shaped	60–120	95%	89–59	—	Bone scaffold application	228
Ca(NO_3_)_2_·4H_2_O and (NH_4_)_2_HPO_4_	Sol–gel	Sr	—	10–11	50	—	200	—	—	115.7	Adsorbent for Cd(ii) removal from wastewater	229
Ca(NO_3_)_2_·6H_2_O and (NH_4_)_2_HPO_4_	Chemical co-precipitation	Zn	—	11	—	Round	—	—	60–41	—	Bone tissue engineering	230
Ca(OH)_2_ and H_3_PO_4_	Wet-chemical precipitation	Eu	—	8	80	Rod-like	100	—	18–13	—	Drug/gene carrier	231
		Fe										
Ca(OH)_2_ and H_3_PO_4_	Hydrothermal	Si	—	—	—	Spherical	—	—	Decrease	—	Biomedical	232
Ca(OH)_2_ and H_3_PO_4_	Microwave-assisted co-precipitation	Al	—	—	Room temp.	Spherical and plate-shaped	61–90	—	35–77	—	Orthopedic application	233

## 4. Discussion

### 4.1 Synthesis methods

As stated before, there are several synthesis methods for HAp, and each of these methods has its advantages and drawbacks, which make them suitable for specific applications. To ensure the large-scale production and practical application of HAp nanoparticles, a simple, environmentally sustainable synthesis method with high-quality nanoparticle yield in significant quantities is essential (Table 7).

**Table 7:** Comparison of synthesis techniques

Methods	Advantages	Limitations	Best application	Ref.
Wet chemical	Simple and low-temperature processing	Lower crystallinity	Photocatalysis	119
Microwave-assisted	Rapid synthesis, energy-efficient, and higher crystallinity	Requires specialized equipment	Drug delivery and rapid HAp production	234
Sol–gel	High purity, homogeneity, and controlled morphology	Long processing time, requires organic solvents	Biomedical application and composite materials	170
Hydrothermal	Lesser energy requirement, high crystallinity, uniform morphology, and tunable porosity	Requires high pressure and long reaction times	Bone tissue engineering and bioactive coatings	235

Hydrothermal synthesis became widely recognized in the 20th century.^235^ Its ability to create high-purity end products, improved morphological control,^236^ and compatibility with large-capacity equipment revived commercial interest.^237^ Similarly, microwave-assisted synthesis is another promising technique because of its uniform heating, faster reaction rates, and pollution-free operation, which result in narrower size distributions, improved crystallinity, and smaller particle sizes.^238^ Also, it is affordable, which increases the appropriateness for production on a wide scale.

### 4.2 Suitable modifier for specific applications

Selection of the optimal combination of synthesis route and modifiers mainly depends on the target application. For photocatalytic activity, the wet chemical method with the organic modifier naphthalene is preferable as it increases the surface area and results in a higher degradation percentage and capacity.^119^ In bone tissue engineering, citric acid is a good option for its ability to control nucleation and crystallinity, and it also produces smaller, rod-shaped particles.^115^ However, the sol–gel method with stearic acid (SA) works better, as mesoporous hydroxyapatite (MPHA) offers high biocompatibility, an increased surface-to-volume ratio, and improved adsorption capability. Stearic acid also contributes to high surface area and porosity, with a pore size of 5.84 nm (BET analysis) and exceptional cytocompatibility, achieving cell viability of up to 83%.^170^

For gene therapy, LPEI is the most suitable modifier, as it improves the strength of biocomposites and produces plate-like morphology, making it a preferred choice.^124^ In drug delivery applications, some modifiers work very well, such as oxalic acid (with a surface area of 89 m^2^ g^−1^), which enhances the porosity and mesoporous structure, making it highly effective.^140^ A non-toxic alternative is serine with the microwave-assisted method, which has a stronger electrostatic effect on the surface of the crystal, resulting in a great impact on crystallization characteristics, which enhances biocompatibility and is promising for drug delivery applications.^146^ Chitosan as a modifier increases pore size (average pore diameter ∼ 38 nm), which makes it particularly useful for slow drug release applications, especially for osteoporosis treatment.^129^ Under hydrothermal conditions, Triton X-100 increases the pore volume and effectively produces a combination of meso- and macropores suitable for catalysis and drug delivery.^180^ For eco-friendly synthesis, caffeine can be considered as it prevents agglomeration of nanoparticles similar to citric acid, and it promotes the formation of well-defined nanorods, with improved shape and size as the concentration increases.^128^ EDTA is beneficial for electrochemical sensing of uric acid and biosensor applications.^146,186^ EDTA is also preferred for synthesizing hydroxyapatite (HAp) with high surface area, uniform microporosity, and strong ion-exchange capacity, and is promising for environmental remediation and nuclear wastewater treatment applications.^168^ In adsorption applications, a combination of cationic–anionic surfactants (CTAB, SDBS, and SDS) increases the surface area, leading to better dye adsorption capacity.^149^

## 5. Challenges and limitations

While modifiers play a crucial role in controlling the morphology, porosity, and surface area of HAp nanomaterials,^153^ their use also presents certain limitations and challenges. One of the primary concerns is the cost associated with modifiers. They can significantly increase the overall expense of the synthesis process. Studies suggested that phase-pure HAp nanorods can be synthesized through simpler, cost-effective routes without the need for hard templates or surfactants,^142^ thereby raising doubt about the necessity of modifiers in certain applications. Some modifiers work better at higher temperatures, for instance, glutamic acid shows lower solubility at room temperature, and solubility increases with higher temperature.^239^ Experimental data showed that the solubility of gallic acid increases from ∼0.72 to ∼29 g per 100 g water for 273 K to 373 K temperature.^240^ Achieving higher temperatures is another cost-intensive factor for a process. Concentration control of modifiers is another significant obstacle. As mentioned before, with increasing concentration, caffeine produces nano-HAp with well-defined characteristics, but higher concentrations can lead to excessive particle growth and secondary agglomeration if not controlled properly.^128^ It emphasizes the importance of optimization to avoid structural inconsistencies and material loss. Some modifiers possess environmental risk due to their potential toxicity. The toxicity of common surfactants like Tergitol NP-10, Triton X-100, and Tween 40 was evaluated using the Microtox® acute toxicity test. According to the findings, all of these surfactants had EC_50_ values less than 100 mg L^−1^, suggesting that they are somewhat toxic and could be dangerous for aquatic life.^241^ Use of surfactants is estimated at over 15 million tonnes per year, and reports indicate that up to 60% (by weight) may be discharged into water bodies. Synthetic surfactants tend to persist in ecosystems due to their low biodegradability, where they can disrupt biological processes, promote eutrophication, and cause foaming in water bodies. Furthermore, their degradation byproducts may exhibit higher toxicity than the original compounds, and they can facilitate the mobilization of other contaminants, such as heavy metals.^242^ Some surfactants, such as SDS, cocamidopropyl betaine (CAPB), have been shown to cause significant cytotoxicity in uncalcined hydroxyapatite (HAp), with cell viability dropping below 70%, compromising biocompatibility.^243^ Therefore, the implementation of modifiers must be evaluated against cost, processing complexity, environmental concerns, and scalability to ensure workable and effective synthesis methodologies for biomedical and industrial use.

## 6. Future recommendations

From the identified limitations, it is clear that we need to explore more eco-friendly substituts to improve the sustainability and versatility of HAp synthesis. One promising approach is using inexpensive, natural modifiers such as tea polyphenols, which can improve the mechanical strength, osteoconductivity, and biocompatibility with necessary porosity and crystallinity for biomedical applications.^244^ Researchers should explore other natural compounds with similar benefits. Another environment-friendly way is the synthesis of HAp from biogenic sources, such as eggshells, fish bones, and mussels,^245,246^ which can minimize waste, cut production costs, and preserve high material purity along with modifiers.^247^ Expanding research on these biogenic HAp syntheses can help with environmental cleanup and sustainable biomedical applications, and it can be a good substitute for traditional synthetic techniques. On top of that, advanced modeling techniques, such as numerical analysis for scaffold fabrication, offer a powerful tool for optimizing sintering temperatures, compaction loads, and microstructural integrity.^248^ These computational approaches can improve the mechanical performance of HAp scaffolds, which may ensure their suitability for clinical applications where structural reliability is vital, and subsequent studies can improve potential weaknesses and optimize system efficiency.

## 7. Conclusion

HAp can be synthesized through various methods; among them, four methods are mostly common: the wet chemical technique, microwave-assisted method, sol–gel method, and hydrothermal method. As the diversity of HAp applications expanded, modifiers such as CTAB, EDTA, amino acids, urea, fatty acids, Triton X-100, polyethylene glycol, citric acid, SDS, *etc.*, were considered by researchers to synthesize HAp particles with a uniform shape, size, and properties. In general, these modifiers control the crystallization process and promote growth in a particular direction. Strong chelating agents such as citric acid and EDTA produce uniform particles as they limit calcium availability effectively. Chelating agents, surfactants, and natural polymers such as chitosan can produce larger and interconnected pores, making nano-HAp highly suitable for biomedical applications, particularly for drug delivery. Currently, researchers are prioritizing eco-friendly modifiers such as caffeine or amino acids. Caffeine works as a stabilizing agent by interacting with the surface of nanoparticles. Amino acids, on the other hand, promote growth in a particular direction by adsorbing on the outer surface of nanocrystals. Notably, the fundamental mechanisms of these modifiers that influence HAp synthesis remain consistent across different synthesis methods. Instead, it is the variation in the concentration of these modifiers and the reaction conditions—such as temperature, pH, and synthesis duration—that primarily drive differences in the resulting HAp structure and morphology. Despite the benefits of modifiers in customizing the morphology of nano-HAp, their use in the synthesis process is relatively unexplored. This is mainly due to the complexity of synthesis, cost, application-specific limitations, and environmental concerns. However, future researchers can focus on eco-friendly, biocompatible modifiers in nano-HAp synthesis to expand applications while addressing cost, versatility, and safety.

## Author contributions

Nahida Sultana Bristy contributed to conceptualization, methodology, analysis, writing – original draft, and writing – review and editing. Md. Kawsar contributed to conceptualization, writing – review and editing, and validation. Md. Sahadat Hossain contributed to supervision, writing – review and editing, and validation.

## Conflicts of interest

The authors have no conflicts to declare.

## Data Availability

Data will be made available on request.

## References

[cit1] Cursaru L. M., Iota M., Piticescu R. M., Tarnita D., Savu S. V., Savu I. D. (2022). *et al.*, Hydroxyapatite from natural sources for medical applications. Materials.

[cit2] Izzetti R., Gennai S., Nisi M., Gulia F., Miceli M., Giuca M. R. (2022). Clinical applications of nano-hydroxyapatite in dentistry. Appl. Sci..

[cit3] Jia X., Shi N., Tang W., Su Z., Chen H., Tang Y. (2022). *et al.*, Nano-hydroxyapatite increased soil quality and boosted beneficial soil microbes. Plant Nano Biol..

[cit4] Avşar C., Gezerman A. O. (2023). An evaluation of phosphogypsum (PG)-derived nanohydroxyapatite (HAP) synthesis methods and waste management as a phosphorus source in the agricultural industry. Mater. Sci..

[cit5] Ibrahim M., Labaki M., Giraudon J. M., Lamonier J. F. (2020). Hydroxyapatite, a multifunctional material for air, water and soil pollution control: a review. J. Hazard. Mater..

[cit6] Balasooriya I. L., Chen J., Korale Gedara S. M., Han Y., Wickramaratne M. N. (2022). Applications of nano hydroxyapatite as adsorbents: a review. Nanomaterials.

[cit7] Pushpalatha C., Gayathri V. S., Sowmya S. V., Augustine D., Alamoudi A., Zidane B. (2023). *et al.*, Nanohydroxyapatite in dentistry: a comprehensive review. Saudi Dent. J..

[cit8] Mateus A. P., Monteiro F. J., Ferraz M. P. (2013). Nanoparticles of hydroxyapatite: preparation, characterization and cellular approach - an overview. Rev. Mutis..

[cit9] Taskin H., Gunes A. (2023). Synthetic nano-hydroxyapatite as an alternative phosphorus source for wheat grown under field conditions. J. Plant Nutr..

[cit10] Fox K., Tran P. A., Tran N. (2012). Recent Advances in Research Applications of Nanophase Hydroxyapatite. ChemPhysChem.

[cit11] Saxena V., Pandey L., Srivatsan T. S. (2021). Nano Hydroxyapatite (nano-HAp): A Potential Bioceramic for Biomedical Applications. Curr. Nanomater..

[cit12] Bhandari N. L., Bista S., Gautam T. R., Bist K., Bhandari G., Subedi S. (2021). *et al.*, An Overview of Synthesis Based Biomedical Applications of Hydroxyapatite Nanomaterials. J. Nepal Chem. Soc..

[cit13] Jaffe W. L., Scott D. F. (1996). Current concepts review-total hip arthroplasty with hydroxyapatite-coated prostheses. J. Bone Jt. Surg..

[cit14] Brown P. W. and Constantz B., Hydroxyapatite and Related Materials, CRC Press, Boca Raton, FL, USA, 1994

[cit15] Thirumalai J. , Introductory chapter: the testament of hydroxyapatite: new prospects in regenerative medicinal treatments, in Hydroxyapatite-Advances in Composite Nanomaterials, Biomedical Applications and its Technological Facets, 2018

[cit16] Sakae T., Nakada H., John P. (2015). LeGeros. Historical Review of Biological Apatite Crystallography. J. Hard Tissue Biol..

[cit17] Jemli Y. E. , Abdelouahdi K., Minh D. P., Barakat A. and Solhy A., Synthesis and Characterization of Hydroxyapatite and Hydroxyapatite-Based Catalysts, Design and Applications of Hydroxyapatite-Based Catalysts, 2022, pp. 19–72

[cit18] Maskowicz D., Maroszek K., Jendrzejewski R., Sawczak M. (2024). Hydroxyapatite Nanocoatings Deposited by Means of Resonant Matrix-Assisted Pulsed Laser Evaporation. Materials.

[cit19] Gu M., Li W., Jiang L., Li X. (2022). Recent progress of rare earth doped hydroxyapatite nanoparticles:
luminescence properties, synthesis and biomedical applications. Acta Biomater..

[cit20] Horiuchi N., Madokoro K., Nozaki K., Nakamura M., Katayama K., Nagai A. (2018). *et al.*, Electrical conductivity of polycrystalline hydroxyapatite and its application to electret formation. Solid State Ionics.

[cit21] Manjubaashini N., Radha G., Balakumar S. (2022). A comprehensive review on functionalized hydroxyapatite nanostructures based gas sensors for environmental pollutant monitoring. Mater. Nanosci..

[cit22] Pinto G., Caira S., Mamone G., Ferranti P., Addeo F., Picariello G. (2014). Fractionation of complex lipid mixtures by hydroxyapatite chromatography for lipidomic purposes. J. Chromatogr. A.

[cit23] Ibrahim M., Labaki M., Giraudon J. M., Lamonier J. F. (2020). Hydroxyapatite, a multifunctional material for air, water and soil pollution control: a review. J. Hazard. Mater..

[cit24] Li Z., Zhou M. m., Lin W. (2014). The Research of Nanoparticle and Microparticle Hydroxyapatite Amendment in Multiple Heavy Metals Contaminated Soil Remediation. J. Nanomater..

[cit25] Sadat-Shojai M., Khorasani M. T., Dinpanah-Khoshdargi E., Jamshidi A. (2013). Synthesis methods for nanosized hydroxyapatite with diverse structures. Acta Biomater..

[cit26] Chou P. Y., Chou Y. C., Lai Y. H., Lin Y. T., Lu C. J., Liu S. J. (2021). Fabrication of Drug-Eluting Nano-Hydroxylapatite Filled Polycaprolactone Nanocomposites Using Solution-Extrusion 3D Printing Technique. Polymers.

[cit27] Yu W., Guo H., Liu Y., Zhou Y., Xiao Y., Bai J. (2025). *et al.*, Development of hydroxyapatite-based Carboxymethylcellulose-Al(III) aerogel beads for efficient and selective defluorination from brick tea infusions. Carbohydr. Polym..

[cit28] Qin D., Zhao Y., Cheng R., Liu Y., Guo S., Sun L., Guo Y., Hao F., Zhao B. (2024). Mussel-inspired immunomodulatory and osteoinductive dual-functional hydroxyapatite nanoplatform for promoting bone regeneration. J. Nanobiotechnol..

[cit29] Vijayan V., Sreekumar S., Ahina K. M., Lakra R., Kiran M. S. (2023). Lanthanum Oxide Nanoparticles Reinforced Collagen κ-Carrageenan Hydroxyapatite Biocomposite as Angio-Osteogenic Biomaterial for In Vivo Osseointegration and Bone Repair. Adv. Biol..

[cit30] Ahmed L. O., Omer R. A. (2024). Hydroxyapatite biomaterials: a comprehensive review of their properties, structures, clinical applications, and producing techniques. Rev. Inorg. Chem..

[cit31] Kareem R. O., Bulut N., Kaygili O. (2024). Hydroxyapatite biomaterials: a comprehensive review of their properties, structures, medical applications, and fabrication methods. J. Chem. Rev..

[cit32] Leventouri T. (2006). Synthetic and biological hydroxyapatites: crystal structure questions. Biomaterials.

[cit33] Bystrov V. S. (2017). Computer studies of hydroxyapatite nanostructures, their features and properties. Math. Biol. Bioinform.

[cit34] Pokhrel S. (2018). Hydroxyapatite: Preparation, Properties and Its Biomedical Applications. Adv. Chem. Eng. Sci..

[cit35] Zhou H., Lee J. (2011). Nanoscale hydroxyapatite particles for bone tissue engineering. Acta Biomater..

[cit36] Fox K., Tran P. A., Tran N. (2012). Recent Advances in Research Applications of Nanophase Hydroxyapatite. ChemPhysChem.

[cit37] Bertinetti L., Tampieri A., Landi E., Ducati C., Midgley P. A., Coluccia S. (2007). *et al.*, Surface Structure, Hydration,
and Cationic Sites of Nanohydroxyapatite: UHR-TEM, IR, and Microgravimetric Studies. J. Phys. Chem. C.

[cit38] Kantharia N., Naik S., Apte S., Kheur M., Kheur S., Kale B. (2014). Nano-hydroxyapatite and its contemporary applications. Bone.

[cit39] Lin K. and Chang J., Structure and properties of hydroxyapatite for biomedical applications, in Hydroxyapatite (HAp) for Biomedical Applications, Woodhead Publishing, 2015, pp. 3–19

[cit40] Pande C. S., Cooper K. P. (2009). Nanomechanics of Hall–Petch relationship in nanocrystalline materials. Prog. Mater. Sci..

[cit41] Wang J., Shaw L. L. (2009). Nanocrystalline hydroxyapatite with simultaneous enhancements in hardness and toughness. Biomaterials.

[cit42] Sun W., Chu C., Wang J., Zhao H. (2007). Comparison of periodontal ligament cells responses to dense and nanophase hydroxyapatite. J. Mater. Sci. Mater. Med..

[cit43] Lin K., Zhou Y., Zhou Y., Qu H., Chen F., Zhu Y. (2011). *et al.*, Biomimetic hydroxyapatite porous microspheres with co-substituted essential trace elements: surfactant-free hydrothermal synthesis, enhanced degradation and drug release. J. Mater. Chem..

[cit44] Wijesinghe W. P. (2014). Overview to hydroxyapatite nanoparticles and their applications. Sciscitator.

[cit45] Mohd Pu'ad N. A. S., Abdul Haq R. H., Mohd Noh H., Abdullah H. Z., Idris M. I., Lee T. C. (2020). Synthesis method of hydroxyapatite: a review. Mater. Today Proc..

[cit46] Dou L., Zhang Y., Sun H. (2018). Advances in Synthesis and Functional Modification of Nanohydroxyapatite. J. Nanomater..

[cit47] Pham T. T. T., Nguyen T. P., Pham T. N., Vu T. P., Tran D. L., Thai H. (2013). *et al.*, Impact of physical and chemical parameters on the hydroxyapatite nanopowder synthesized by chemical precipitation method. Adv. Nat. Sci. Nanosci. Nanotechnol..

[cit48] Karunakaran G., Cho E. B., Kumar G. S., Kolesnikov E., Sudha K. G., Mariyappan K. (2022). *et al.*, Citric Acid-Mediated Microwave-Hydrothermal Synthesis of Mesoporous F-Doped HAp Nanorods from Bio-Waste for Biocidal Implant Applications. Nanomaterials.

[cit49] Sun X. D., Liu T. T., Wang Q. Q., Zhang J., Cao M. S. (2023). Surface Modification and Functionalities for Titanium Dental Implants. ACS Biomater. Sci. Eng..

[cit50] Han Y., Li S., Wang X., Chen X. (2004). Synthesis and sintering of nanocrystalline hydroxyapatite powders by citric acid sol–gel combustion method. Mater. Res. Bull..

[cit51] Yan L., Li Y., Deng Z. X., Zhuang J., Sun X. (2001). Surfactant-assisted hydrothermal synthesis of hydroxyapatite nanorods. Int. J. Inorg. Mater..

[cit52] Liu J., Li K., Wang H., Zhu M., Yan H. (2004). Rapid formation of hydroxyapatite nanostructures by microwave irradiation. Chem. Phys. Lett..

[cit53] Wang A., Yin H., Liu D., Wu H., Wada Y., Ren M. (2007). *et al.*, Effects of organic modifiers on the size-controlled synthesis of hydroxyapatite nanorods. Appl. Surf. Sci..

[cit54] Mohd Pu'ad N. A. S., Abdul Haq R. H., Mohd Noh H., Abdullah H. Z., Idris M. I., Lee T. C. (2020). Synthesis method of hydroxyapatite: a review. Mater. Today Proc..

[cit55] Anandan D., Jaiswal A. K. (2024). Synthesis methods of hydroxyapatite and biomedical applications: an updated review. J. Aust. Ceram. Soc..

[cit56] Sadat-Shojai M., Khorasani M. T., Dinpanah-Khoshdargi E., Jamshidi A. (2013). Synthesis methods for nanosized hydroxyapatite with diverse structures. Acta Biomater..

[cit57] Monmaturapoj N. (2008). Nano-size hydroxyapatite powders preparation by wet-chemical precipitation route. J. Met. Mater. Miner..

[cit58] Yelten-Yilmaz A., Yilmaz S. (2018). Wet chemical precipitation synthesis of hydroxyapatite (HA) powders. Ceram. Int..

[cit59] Abidi S. S. A., Murtaza Q. (2014). Synthesis and Characterization of Nano-hydroxyapatite Powder Using Wet Chemical Precipitation Reaction. J. Mater. Sci. Technol..

[cit60] Chandrasekar A., Sagadevan S., Dakshnamoorthy A. (2013). Synthesis and characterization of nano-hydroxyapatite (n-HAP) using the wet chemical technique. Int. J. Phys. Sci..

[cit61] Wang P., Li C., Gong H., Jiang X., Wang H., Li K. (2010). Effects of synthesis conditions on the morphology of hydroxyapatite nanoparticles produced by wet chemical process. Powder Technol..

[cit62] Eslami H., Tahriri M., Bakhshi F. (2010). Synthesis and characterization of nanocrystalline hydroxyapatite obtained by the wet chemical technique. Mater. Sci..

[cit63] Kalaiselvi V., Mathammal R., Anitha P. (2017). Synthesis and Characterization of Hydroxyapatite Nanoparticles using Wet Chemical Method. Int. J. Adv. Sci. Eng..

[cit64] Gentile P., Wilcock C. J., Miller C. A., Moorehead R., Hatton P. V. (2015). Process Optimisation to Control the Physico-Chemical Characteristics of Biomimetic Nanoscale Hydroxyapatites Prepared Using Wet Chemical Precipitation. Materials.

[cit65] Awan A. A., Liaqat U., Hussain Z. (2023). The effect of pH on the morphological transformation of nanocrystalline hydroxyapatite during wet chemical synthesis. J. Korean Ceram. Soc..

[cit66] Council NR , Sciences D on E and P, Board NMA, Systems C on E and T, Technology C on MP of MAEI, Microwave Processing of Materials, National Academies Press, 1994, p. 165

[cit67] Cai Z., Wang X., Zhang Z., Han Y., Luo J., Huang M. (2019). *et al.*, Large-scale and fast synthesis of nano-hydroxyapatite powder by a microwave-hydrothermal method. RSC Adv..

[cit68] Mishra V. K., Rai S. B., Asthana B. P., Parkash O., Kumar D. (2014). Effect of annealing on nanoparticles of hydroxyapatite synthesized via microwave irradiation: structural and spectroscopic studies. Ceram. Int..

[cit69] Sabu U., Logesh G., Rashad M., Joy A., Balasubramanian M. (2019). Microwave assisted synthesis of biomorphic hydroxyapatite. Ceram. Int..

[cit70] Shaban N. Z., Kenawy M. Y., Taha N. A., Abd El-Latif M. M., Ghareeb D. A. (2021). Synthesized Nanorods Hydroxyapatite by Microwave-Assisted Technology for In vitro Osteoporotic Bone Regeneration through Wnt/β-Catenin Pathway. Materials.

[cit71] Natarajan N., Prabasheela B. (2022). Microwave abetted preparation of nano-hydroxyapatite for orthopedic application with in vitro evidence. AIP Conf. Proc..

[cit72] Hassan M. N., Mahmoud M. M., El-Fattah A. A., Kandil S. (2016). Microwave-assisted preparation of Nano-hydroxyapatite for bone substitutes. Ceram. Int..

[cit73] Lerner E., Sarig S., Azoury R. (1991). Enhanced maturation of
hydroxyapatite from aqueous solutions using microwave irradiation. J. Mater. Sci. Mater. Med..

[cit74] Vaidhyanathan B., Rao K. J. (1996). Rapid microwave assisted synthesis of hydroxyapatite. Bull. Mater. Sci..

[cit75] Sánchez-Campos D., Reyes Valderrama M. I., López-Ortíz S., Salado-Leza D., Fernández-García M. E., Mendoza-Anaya D. (2021). *et al.*, Modulated Monoclinic Hydroxyapatite: The Effect of pH in the Microwave Assisted Method. Minerals.

[cit76] Alshemary A. Z., Goh Y. F., Akram M., Razali I. R., Abdul Kadir M. R., Hussain R. (2013). Microwave assisted synthesis of nano sized sulphate doped hydroxyapatite. Mater. Res. Bull..

[cit77] Sözügeçer S., Bayramgil N. P. (2021). Preparation and characterization of polyacrylic acid-hydroxyapatite nanocomposite by microwave-assisted synthesis method. Heliyon.

[cit78] Fu L. H., Liu Y. J., Ma M. G., Zhang X. M., Xue Z. M., Zhu J. F. (2016). Microwave-Assisted Hydrothermal Synthesis of Cellulose/Hydroxyapatite Nanocomposites. Polymers.

[cit79] Tolga Demirtaş T., Kaynak G., Gümüşderelioğlu M. (2015). Bone-like hydroxyapatite precipitated from 10×SBF-like solution by microwave irradiation. Mater. Sci. Eng., C.

[cit80] López-Macipe A., Gómez-Morales J., Rodríguez-Clemente R. (1998). Nanosized Hydroxyapatite Precipitation from Homogeneous Calcium/Citrate/Phosphate Solutions Using Microwave and Conventional Heating. Adv. Mater..

[cit81] Kalita S. J., Verma S. (2010). Nanocrystalline hydroxyapatite bioceramic using microwave radiation: synthesis and characterization. Mater. Sci. Eng., C.

[cit82] Han J. K., Song H. Y., Saito F., Lee B. T. (2006). Synthesis of high purity nano-sized hydroxyapatite powder by microwave-hydrothermal method. Mater. Chem. Phys..

[cit83] Demazeau G. (2010). Review. Solvothermal Processes: Definition, Key Factors Governing the Involved Chemical Reactions and New Trends. Z. Naturforsch., B:J. Chem. Sci..

[cit84] Cao J. M., Feng J., Deng S. G., Chang X., Wang J., Liu J. S. (2005). *et al.*, Microwave-assisted solid-state synthesis of hydroxyapatite nanorods at room temperature. J. Mater. Sci..

[cit85] Zyman Z., Goncharenko A., Rokhmistrov D., Epple M. (2011). Nanocrystalline calcium-deficient hydroxyapatite prepared by a microwave-assisted solvent-free reaction. Mater. Werkst..

[cit86] Jalota S., Tas A. C., Bhaduri S. B. (2004). Microwave-assisted synthesis of calcium phosphate nanowhiskers. J. Mater. Res..

[cit87] Wagner D., Eisenmann K., Kalinoski A., Bhaduri S. (2013). A Microwave Assisted Solution Combustion Synthesis (MASCS) to Produce Europium Doped Calcium Phosphate Nanowhiskers for Bioimaging Applications. Acta Biomater..

[cit88] Liang T., Qian J., Yuan Y., Liu C. (2013). Synthesis of mesoporous hydroxyapatite nanoparticles using a template-free sonochemistry-assisted microwave method. J. Mater. Sci..

[cit89] Zou Z., Lin K., Chen L., Chang J. (2012). Ultrafast synthesis and characterization of carbonated hydroxyapatite nanopowders via sonochemistry-assisted microwave process. Ultrason. Sonochem..

[cit90] Yang Z., Jiang Y., Wang Y., Ma L., Li F. (2004). Preparation and thermal stability analysis of hydroxyapatite derived from the precipitation process and microwave irradiation method. Mater. Lett..

[cit91] Bokov D., Turki Jalil A., Chupradit S., Suksatan W., Javed Ansari M., Shewael I. H. (2021). *et al.*, Nanomaterial by Sol-Gel Method: Synthesis and Application. Adv. Mater. Sci. Eng..

[cit92] Fathi M. H., Hanifi A. (2007). Evaluation and characterization of nanostructure hydroxyapatite powder prepared by simple sol–gel method. Mater. Lett..

[cit93] Bakan F., Laçin O., Sarac H. (2013). A novel low temperature sol–gel synthesis process for thermally stable nano crystalline hydroxyapatite. Powder Technol..

[cit94] Yusoff Y. M. , Salimi M. N. and Anuar A., Preparation of hydroxyapatite nanoparticles by sol-gel method with optimum processing parameters, in AIP Conference Proceedings, 2015, ch. 1, vol. 1660

[cit95] Agrawal K., Singh G., Puri D., Prakash S. (2011). Synthesis and Characterization of Hydroxyapatite Powder by Sol-Gel Method for Biomedical Application. J. Miner. Mater. Char. Eng..

[cit96] Hosseini B., Mirhadi S. M., Mehrazin M., Yazdanian M., Alantar Motamedi M. R. (2017). Synthesis of nanocrystalline hydroxyapatite using eggshell and trimethyl phosphate. Trauma Mon..

[cit97] Kim I. S., Kumta P. N. (2004). Sol–gel synthesis and characterization of nanostructured hydroxyapatite powder. Mater. Sci. Eng., B.

[cit98] Jaafar A., Hecker C., Árki P., Joseph Y. (2020). Sol-Gel Derived Hydroxyapatite Coatings for Titanium Implants: A Review. Bioengineering.

[cit99] Choi G., Choi A. H., Evans L. A., Akyol S., Ben-Nissan B. (2020). A review: recent advances in sol-gel-derived hydroxyapatite nanocoatings for clinical applications. J. Am. Ceram. Soc..

[cit100] Bilton M., Brown A. P., Milne S. J. (2010). Sol-gel synthesis and characterisation of nano-scale hydroxyapatite. J. Phys.:Conf. Ser..

[cit101] Türk S., Altınsoy İ., Çelebi Efe G., Ipek M., Özacar M., Bindal C. (2019). Effect of Solution and Calcination Time on Sol-gel Synthesis of Hydroxyapatite. J. Bionic. Eng..

[cit102] Jaafar A., Schimpf C., Mandel M., Hecker C., Rafaja D., Krüger L. (2022). *et al.*, Sol–gel derived hydroxyapatite coating on titanium implants: optimization of sol–gel process and engineering the interface. J. Mater. Res..

[cit103] Fiume E., Magnaterra G., Rahdar A., Verné E., Baino F. (2021). Hydroxyapatite for Biomedical Applications: A Short Overview. Ceramics.

[cit104] Earl J. S., Wood D. J., Milne S. J. (2006). Hydrothermal synthesis of hydroxyapatite. J. Phys.:Conf. Ser..

[cit105] Canu G., Buscaglia V. (2017). Hydrothermal synthesis of strontium titanate: thermodynamic considerations, morphology control and crystallisation mechanisms. CrystEngComm.

[cit106] Gan Y. X., Jayatissa A. H., Yu Z., Chen X., Li M. (2020). Hydrothermal Synthesis of Nanomaterials. J. Nanomater..

[cit107] Zhang X., Vecchio K. S. (2007). Hydrothermal synthesis of hydroxyapatite rods. J. Cryst. Growth.

[cit108] Neira I. S., Guitián F., Taniguchi T., Watanabe T., Yoshimura M. (2008). Hydrothermal synthesis of hydroxyapatite whiskers with sharp faceted hexagonal morphology. J. Mater. Sci..

[cit109] Jinawath S., Pongkao D., Yoshimura M. (2002). Hydrothermal synthesis of hydroxyapatite from natural source. J. Mater. Sci. Mater. Med..

[cit110] Nayak A. K. (2010). Hydroxyapatite synthesis methodologies: an overview. Int. J. ChemTech Res..

[cit111] Fatimah M., Shaaban A., Seliman S. (2012). Overview: Process Parameters for Hydrothermal Synthesis of Hydroxyapatite. J. Adv. Manuf. Technol. (JAMT)..

[cit112] Komarneni S. (2003). Nanophase materials by hydrothermal, microwave-hydrothermal and microwave-solvothermal methods. Curr. Sci..

[cit113] Shi W., Song S., Zhang H. (2013). Hydrothermal synthetic strategies of inorganic semiconducting nanostructures. Chem. Soc. Rev..

[cit114] Abidi S. S. A., Murtaza Q. (2014). Synthesis and Characterization of Nano-hydroxyapatite Powder Using Wet Chemical Precipitation Reaction. J. Mater. Sci. Technol..

[cit115] Aneem T. H., Saha S. K., Jahan R. A., Wong S. Y., Li X., Arafat M. T. (2019). Effects of organic modifiers and temperature on the synthesis of biomimetic carbonated hydroxyapatite. Ceram. Int..

[cit116] Gonsalves J. K. M. C., Ferro J. N. S., Barreto E. O., Nunes R. S., Valerio M. E. G. (2016). Influence of concentration of hydroxyapatite surface modifier agent on bioactive composite characteristics. Ceram. Int..

[cit117] Bricha M., Belmamouni Y., Essassi E. M., Ferreira J. M. F., Mabrouk K. E. (2012). Surfactant-Assisted Hydrothermal Synthesis of Hydroxyapatite Nanopowders. J. Nanosci. Nanotechnol..

[cit118] Neelgund G. M., Oki A. (2022). Photocatalytic activity of hydroxyapatite deposited graphene nanosheets under illumination to sunlight. Mater. Res. Bull..

[cit119] Kawsar M., Hossain M. S., Tabassum S., Mohammed Bahadur N., Ahmed S. (2024). Different solvents and organic modifiers for the control of crystallographic parameters in nano-crystallite hydroxyapatite for amplification of photocatalytic activity. Nanoscale Adv..

[cit120] Barabás R., Czikó M., Dékány I., Bizo L., Bogya E. S. (2013). Comparative study of particle size analysis of hydroxyapatite-based nanomaterials. Chem. Pap..

[cit121] Gonsalves J. K. M. C., Ferro J. N. S., Barreto E. O., Nunes R. S., Valerio M. E. G. (2016). Influence of concentration of hydroxyapatite surface modifier agent on bioactive composite characteristics. Ceram. Int..

[cit122] Martins M. A., Santos C., Almeida M. M., Costa M. E. V. (2008). Hydroxyapatite micro- and nanoparticles: nucleation and growth mechanisms in the presence of citrate species. J. Colloid Interface Sci..

[cit123] Wang A., Liu D., Yin H., Wu H., Wada Y., Ren M. (2007). *et al.*, Size-controlled synthesis of hydroxyapatite nanorods by chemical precipitation in the presence of organic modifiers. Mater. Sci. Eng., C.

[cit124] Timpu D., Sacarescu L., Vasiliu T., Dinu M. V., David G. (2020). Surface cationic functionalized nano-hydroxyapatite – preparation, characterization, effect of coverage on properties and related applications. Eur. Polym. J..

[cit125] Reddy V. S., Shiva S., Manikantan S., Ramakrishna S. (2024). Pharmacology of caffeine and its effects on the human body. Eur. J. Med. Chem. Rep..

[cit126] Mishra V. K., Bhattacharjee B. N., Kumar D., Rai S. B., Parkash O. (2016). Effect of a chelating agent at different pH on the spectroscopic and structural properties of microwave derived hydroxyapatite nanoparticles: a bone mimetic material. New J. Chem..

[cit127] Mary A. A., Ansari A. T., Subramanian R. (2020). Caffeine-mediated synthesis of CuO nanoparticles: characterization, morphology changes, and bactericidal activity. Inorg. Nano-Met. Chem..

[cit128] Subramanian R., Murugan P., Chinnadurai G., Ponmurugan K., Al-Dhabi N. A. (2020). Experimental studies on caffeine mediated synthesis of hydroxyapatite nanorods and their characterization. Mater. Res. Express.

[cit129] Absalan F., Sadjadi M. S., Farhadyar N., Sadr M. H. (2020). Synthesis of Mesoporous Hydroxyapatite with Controlled Pore Size Using the Chitosan as an Organic Modifier: Investigating the Effect of the Weight Ratio and pH Value of Chitosan on the Structural and Morphological Properties. J. Inorg. Organomet. Polym. Mater..

[cit130] Wang A., Yin H., Liu D., Wu H., Wada Y., Ren M. (2007). *et al.*, Effects of organic modifiers on the size-controlled synthesis of hydroxyapatite nanorods. Appl. Surf. Sci..

[cit131] Neves J. G., Navarro Da Rocha D., Lopes C. C., Prado Da Silva M. H., Sinhoreti M. A. C., Correr-Sobrinho L. (2021). *et al.*, Effect of pH level and calcination on the production of calcium phosphates by acidic route of wet precipitation. Cerâmica.

[cit132] Azzaoui K., Jodeh S., Mejdoubi E., Hammouti B., Taleb M., Ennabety G. (2023). *et al.*, Synthesis of hydroxyapatite/polyethylene glycol 6000 composites by novel dissolution/precipitation method: optimization of the adsorption process using a factorial design: DFT and molecular dynamic. BMC Chem..

[cit133] Muthu D., Suresh Kumar G., Gowri M., Prasath M., Viswabaskaran V., Kattimani V. S. (2022). *et al.*, Rapid synthesis of eggshell derived hydroxyapatite with nanoscale characteristics for biomedical applications. Ceram. Int..

[cit134] Römer I., Briffa S. M., Dasilva Y. A. R., Hapiuk D., Trouillet V., Palmer R. E. (2019). *et al.*, Impact of particle size, oxidation state and capping agent of different cerium dioxide nanoparticles on the phosphate-induced transformations at different pH and concentration. PLoS One.

[cit135] Reddy D. A., Murali G., Vijayalakshmi R. P., Reddy B. K. (2011). Room-temperature ferromagnetism in EDTA capped Cr-doped ZnS nanoparticles. Appl. Phys. A.

[cit136] Anderson S. D., Gwenin V. V., Gwenin C. D. (2019). Magnetic Functionalized Nanoparticles for Biomedical, Drug Delivery and Imaging Applications. Nanoscale Res. Lett..

[cit137] Kalita S. J., Verma S. (2010). Nanocrystalline hydroxyapatite bioceramic using microwave radiation: synthesis and characterization. Mater. Sci. Eng., C.

[cit138] Ruban Kumar A., Kalainathan S., Saral A. M. (2010). Microwave assisted synthesis of hydroxyapatite nano strips. Cryst. Res. Technol..

[cit139] Mishra V. K., Bhattacharjee B. N., Kumar D., Rai S. B., Parkash O. (2016). Effect of a chelating agent at different pH on the spectroscopic and structural properties of microwave derived hydroxyapatite nanoparticles: a bone mimetic material. New J. Chem..

[cit140] Karunakaran G., Kumar G. S., Cho E. B., Sunwoo Y., Kolesnikov E., Kuznetsov D. (2019). Microwave-assisted hydrothermal synthesis of mesoporous carbonated hydroxyapatite with tunable nanoscale characteristics for biomedical applications. Ceram. Int..

[cit141] Gopi D., Bhalaji P. R., Prakash V. C. A., Ramasamy A. K., Kavitha L., Ferreira J. M. F. (2011). An effective and facile synthesis of hydroxyapatite powders using oxalic acid–ethylene glycol mixture. Curr. Appl. Phys..

[cit142] Singh G., Jolly S. S., Singh R. P. (2020). Investigation of surfactant role in synthesis of hydroxyapatite nanorods under microwave and hydrothermal conditions. Mater. Today Proc..

[cit143] Sánchez-Campos D., Mendoza-Anaya D., Reyes-Valderrama M. I., Esteban-Gómez S., Rodríguez-Lugo V. (2020). Cationic surfactant at high pH in microwave HAp synthesis. Mater. Lett..

[cit144] Gopi D., Indira J., Nithiya S., Kavitha L., Mudali U. K., Kanimozhi K. (2013). Influence of surfactant concentration on nanohydroxyapatite growth. Bull. Mater. Sci..

[cit145] Kar S., Kaur T., Thirugnanam A. (2016). Microwave-assisted synthesis of porous chitosan–modified montmorillonite–hydroxyapatite composite scaffolds. Int. J. Biol. Macromol..

[cit146] Chen J., Liu J., Deng H., Yao S., Wang Y. (2020). Regulatory synthesis and characterization of hydroxyapatite nanocrystals by a microwave-assisted hydrothermal method. Ceram. Int..

[cit147] Kalaiselvi V., Mathammal R., Vijayakumar S., Vaseeharan B. (2018). Microwave assisted green synthesis of hydroxyapatite nanorods using *Moringa oleifera* flower extract and its antimicrobial applications. Int. J. Vet. Sci. Med..

[cit148] Liu J., Li K., Wang H., Zhu M., Yan H. (2004). Rapid formation of hydroxyapatite nanostructures by microwave irradiation. Chem. Phys. Lett..

[cit149] Sharma M., Mishra A., Mehta A., Choudhury D., Basu S. (2018). Effect of Surfactants on the structure and adsorption efficiency of hydroxyapatite nanorods. J. Nanosci. Nanotechnol..

[cit150] Rameshbabu N., Kumar T. S. S., Rao K. P. (2010). Influence of microwave power, irradiation time and polymeric additions on synthesis of nanocrystalline hydroxyapatite. Mater. Res. Innov..

[cit151] Kanchana P., Sekar C. (2014). EDTA assisted synthesis of hydroxyapatite nanoparticles for electrochemical sensing of uric acid. Mater. Sci. Eng., C.

[cit152] Muthu D., Kumar G. S., Kattimani V. S., Viswabaskaran V., Girija E. K. (2020). Optimization of a lab scale and pilot scale conversion of eggshell biowaste into hydroxyapatite using microwave reactor. Ceram. Int..

[cit153] Amer W., Abdelouahdi K., Ramananarivo H. R., Zahouily M., Fihri A., Djessas K. (2013). *et al.*, Microwave-assisted synthesis of mesoporous nano-hydroxyapatite using surfactant templates. CrystEngComm.

[cit154] Kumar G. S., Thamizhavel A., Girija E. K. (2012). Microwave conversion of eggshells into flower-like hydroxyapatite nanostructure for biomedical applications. Mater. Lett..

[cit155] Ali A. F., Alrowaili Z. A., El-Giar E. M., Ahmed M. M., El-Kady A. M. (2021). Novel green synthesis of hydroxyapatite uniform nanorods via microwave-hydrothermal route using licorice root extract as template. Ceram. Int..

[cit156] Singh G., Jolly S. S., Singh R. P. (2020). Investigation of surfactant role in synthesis of hydroxyapatite nanorods under microwave and hydrothermal conditions. Mater. Today Proc..

[cit157] Karunakaran G., Cho E. B., Kumar G. S., Kolesnikov E., Karpenkov D. Y., Gopinathan J. (2019). *et al.*, Sodium dodecyl sulfate mediated microwave synthesis of biocompatible superparamagnetic mesoporous hydroxyapatite nanoparticles using black Chlamys varia seashell as a calcium source for biomedical applications. Ceram. Int..

[cit158] Karunakaran G., Cho E. B., Kumar G. S., Kolesnikov E., Sudha K. G., Mariyappan K. (2022). *et al.*, Citric Acid-Mediated Microwave-Hydrothermal Synthesis of Mesoporous F-Doped HAp Nanorods from Bio-Waste for Biocidal Implant Applications. Nanomaterials.

[cit159] Sánchez-Campos D., Mendoza-Anaya D., Reyes-Valderrama M. I., Esteban-Gómez S., Rodríguez-Lugo V. (2020). Cationic surfactant at high pH in microwave HAp synthesis. Mater. Lett..

[cit160] Kumar G. S., Girija E. K., Thamizhavel A., Yokogawa Y., Kalkura S. N. (2010). Synthesis and characterization of bioactive hydroxyapatite–calcite nanocomposite for biomedical applications. J. Colloid Interface Sci..

[cit161] Li Y., Weng W. (2008). Surface modification of hydroxyapatite by stearic acid: characterization and in vitro behaviors. J. Mater. Sci. Mater. Med..

[cit162] Anita L. J., Sundareswari M., Ravichandran K., Bavani Latha M., Sagadevan S., Johan M. R. B. (2019). Tailoring the morphological features of sol–gel synthesized mesoporous hydroxyapatite using fatty acids as an organic modifier. RSC Adv..

[cit163] Jayakodi S., Shanmugam R., Pandian E., Govindasamy M., Asiri J. M., Yadav K. K., Ryeol Choi J. (2024). Controlling pore size during the synthesis of hydroxyapatite nanoparticles using CTAB by the sol–gel hydrothermal method and their biological activities. Nanotechnol. Rev..

[cit164] Ruban Kumar A., Kalainathan S. (2010). Sol–gel synthesis of nanostructured hydroxyapatite powder in presence of polyethylene glycol. Phys. B.

[cit165] Li C. (2009). Crystalline behaviors of hydroxyapatite in the neutralized reaction with different citrate additions. Powder Technol..

[cit166] Martins M. A., Santos C., Almeida M. M., Costa M. E. V. (2008). Hydroxyapatite micro- and nanoparticles: nucleation and growth mechanisms in the presence of citrate species. J. Colloid Interface Sci..

[cit167] Baladi M., Amiri M., Mohammadi P., Mahdi K. S., Golshani Z., Razavi R. (2023). *et al.*, Green sol–gel synthesis of hydroxyapatite nanoparticles using lemon extract as capping agent and investigation of its anticancer activity against human cancer cell lines (T98, and SHSY5). Arabian J. Chem..

[cit168] El-Din A. F. T., Elshehy E. A., El-Khouly M. E. (2018). Cellulose acetate/EDTA-chelator assisted synthesis of ordered mesoporous HAp microspheres for efficient removal of radioactive species from seawater. J. Environ. Chem. Eng..

[cit169] Papynov E. K., Shichalin O. O., Apanasevich V. I., Portnyagin A. S., Yu M. V., Yu B. I. (2020). *et al.*, Sol-gel (template) synthesis of osteoplastic CaSiO3/HAp powder biocomposite: “in vitro” and “in vivo” biocompatibility assessment. Powder Technol..

[cit170] Lett J. A., Sundareswari M., Ravichandran K., Latha M. B., Sagadevan S., MohdR B. J. (2019). Tailoring the morphological features of sol–gel synthesized mesoporous hydroxyapatite using fatty acids as an organic modifier. RSC Adv..

[cit171] Yousefi K. , Zebarjad S. M. and Khaki J. V., Effect of polyethylene glycol and acetic acid on morphology of nanoparticle hydroxyapatite synthesized by sol–gel, 2012

[cit172] Velu G., Gopal B. (2009). Preparation of Nanohydroxyapatite by a Sol–Gel Method Using Alginic Acid as a Complexing Agent. J. Am. Ceram. Soc..

[cit173] Dou Y., Cai S., Ye X., Xu G., Hu H., Ye X. (2012). Preparation of mesoporous hydroxyapatite films used as biomaterials via sol–gel technology. J. Sol-Gel Sci. Technol..

[cit174] Klinkaewnarong J., Utara S. (2017). Preparation and characterization of nanohydroxyapatite by modified sol-gel method with natural rubber latex as a templating agent. Inorg. Nano-Met. Chem..

[cit175] Gonzalez-McQuire R., Chane-Ching J. Y., Vignaud E., Lebugle A., Mann S. (2004). Synthesis and characterization of amino acid-functionalized hydroxyapatite nanorods. J. Mater. Chem..

[cit176] Liu H., Qin J., Li Y., Cui H., Tang H., Yang X. (2012). Hydrothermal synthesis and characterisation of glutamine-modified rod-like hydroxyapatite nanoparticles. Micro Nano Lett..

[cit177] Aida T. M., Yamagata T., Watanabe M., Smith R. L. (2010). Depolymerization of sodium alginate under hydrothermal conditions. Carbohydr. Polym..

[cit178] Wang Y., Ren X., Ma X., Su W., Zhang Y., Sun X. (2015). *et al.*, Alginate-Intervened Hydrothermal Synthesis of Hydroxyapatite Nanocrystals with Nanopores. Cryst. Growth Des..

[cit179] Balakrishnan S., Rajendran A., Kulandaivelu R., Nellaiappan S. N. T. S. (2019). Saponin-mediated synthesis of hydroxyapatite by hydrothermal method: characteristics, bioactivity, and antimicrobial behavior. J. Aust. Ceram. Soc..

[cit180] Iyyappan E., Wilson P., Sheela K., Ramya R. (2016). Role of triton X-100 and hydrothermal treatment on the morphological features of nanoporous hydroxyapatite nanorods. Mater. Sci. Eng., C.

[cit181] Wang A., Yin H., Liu D., Wu H., Ren M., Jiang T. (2007). *et al.*, Size-controlled synthesis of hydroxyapatite nanorods in the presence of organic modifiers. Mater. Lett..

[cit182] Wang A., Yin H., Liu D., Wu H., Wada Y., Ren M. (2007). *et al.*, Effects of organic modifiers on the size-controlled synthesis of hydroxyapatite nanorods. Appl. Surf. Sci..

[cit183] Sahebali M., Sedigheh J. (2009). Synthesis of hydroxyapatite nanostructure by hydrothermal condition for biomedical application. Iran. J. Pharm. Sci..

[cit184] Zhang H. b., Zhou K. c., Li Z. y., Huang S. p., Zhao Y. z. (2009). Morphologies of hydroxyapatite nanoparticles adjusted by organic additives in hydrothermal synthesis. J. Cent. South Univ. Technol..

[cit185] Lak A., Mazloumi M., Mohajerani M., Kajbafvala A., Zanganeh S., Arami H. (2008). *et al.*, Self-Assembly of Dandelion-Like Hydroxyapatite Nanostructures Via Hydrothermal Method. J. Am. Ceram. Soc..

[cit186] Kawsar M., Hossain M. S., Tabassum S., Islam D., Mohammed Bahadur N., Ahmed S. (2024). Crystal structure modification of nano-hydroxyapatite using organic modifiers and hydrothermal technique. RSC Adv..

[cit187] Wang A., Yin H., Liu D., Wu H., Ren M., Jiang T. (2007). *et al.*, Size-controlled synthesis of hydroxyapatite nanorods in the presence of organic modifiers. Mater. Lett..

[cit188] Abdel-Aal E., Abdel-Ghafar H., El-Sayed D., Ewais E. (2022). Synthesis of High Hardness Hydroxyapatite Particles using Surfactant Assisted Hydrothermal Method. Int. J. Mater. Technol. Innov..

[cit189] Bricha M., Belmamouni Y., Essassi E. M., Ferreira J. M. F., Mabrouk K. E. (2012). Surfactant-Assisted Hydrothermal Synthesis of Hydroxyapatite Nanopowders. J. Nanosci. Nanotechnol..

[cit190] Taheri M. M., Abdul Kadir M. R., Shokuhfar T., Hamlekhan A., Assadian M., Shirdar M. R. (2015). *et al.*, Surfactant-assisted hydrothermal synthesis of fluoridated hydroxyapatite nanorods. Ceram. Int..

[cit191] Dou Y. , Cao F., Li Y. B. and Li D. X., In hydrothermal synthesis of hydroxyapatite microspheres with polyvinylpyrrolidone as template, The 7th National Conference on Functional Materials and Applications, ed. G. M. Zhao, 2010, pp. 286–290

[cit192] Buitrago-Vásquez M., Ossa-Orozco C. P. (2018). Hydrothermal synthesis of hydroxyapatite nanorods using a fruit extract template. DYNA.

[cit193] Sneha M., Sundaram N. M., Kandaswamy A. (2016). A Novel Hydrothermal Synthesis of Hydroxyapatite Nanotubes using Ceiba pentandra (Kapok) as Template for Biomedical Applications. Dig. J. Nanomater. Biostruct..

[cit194] Salarian M., Solati-Hashjin M., Shafiei S. S., Goudarzi A., Salarian R., Nemati A. (2009). Surfactant-assisted synthesis and characterization of hydroxyapatite nanorods under hydrothermal conditions. Mater. Sci..

[cit195] Li Y., Tjandra W., Tam K. C. (2008). Synthesis and characterization of nanoporous hydroxyapatite using cationic surfactants as templates. Mater. Res. Bull..

[cit196] Wang Y., Zhang S., Wei K., Zhao N., Chen J., Wang X. (2006). Hydrothermal synthesis of hydroxyapatite nanopowders using cationic surfactant as a template. Mater. Lett..

[cit197] Arce H., Montero M. L., Sáenz A., Castaño V. M. (2004). Effect of pH and temperature on the formation of hydroxyapatite at low temperatures by decomposition of a Ca–EDTA complex. Polyhedron.

[cit198] Radulescu D. E., Vasile O. R., Andronescu E., Ficai A. (2023). Latest research of doped hydroxyapatite for bone tissue engineering. Int. J. Mol. Sci..

[cit199] Mariappan A., Pandi P., Beula Rani K. R., Rajeswarapalanichamy, Neyvasagam K. (2022). Study of the photocatalytic and antibacterial effect of Zn and Cu doped hydroxyapatite. Inorg. Chem. Commun..

[cit200] Karunakaran G., Cho E. B., Kumar G. S., Kolesnikov E., Janarthanan G., Pillai M. M. (2020). *et al.*, Mesoporous Mg-doped hydroxyapatite nanorods prepared from bio-waste blue mussel shells for implant applications. Ceram. Int..

[cit201] Bodhak S., Bose S., Bandyopadhyay A. (2011). Bone cell–material interactions on metal-ion doped polarized hydroxyapatite. Mater. Sci. Eng., C.

[cit202] Jacobs A., Gaulier M., Duval A., Renaudin G. (2019). Silver Doping Mechanism in Bioceramics—From Ag+: Doped HAp to Ag°/BCP Nanocomposite. Crystals.

[cit203] Paluszkiewicz C., Ślósarczyk A., Pijocha D., Sitarz M., Bućko M., Zima A. (2010). *et al.*, Synthesis, structural properties and thermal stability of Mn-doped hydroxyapatite. J. Mol. Struct..

[cit204] Eslami H., Solati-Hashjin M., Tahriri M. (2009). The comparison of powder characteristics and physicochemical, mechanical and biological properties between nanostructure ceramics of hydroxyapatite and fluoridated hydroxyapatite. Mater. Sci. Eng., C.

[cit205] Arcos D., Vallet-Regí M. (2020). Substituted hydroxyapatite coatings of bone implants. J. Mater. Chem. B.

[cit206] Liu X., Mou Y., Wu S., Man H. C. (2013). Synthesis of silver-incorporated hydroxyapatite nanocomposites for antimicrobial implant coatings. Appl. Surf. Sci..

[cit207] Devanand Venkatasubbu G., Ramasamy S., Avadhani G. S., Palanikumar L., Kumar J. (2012). Size-mediated cytotoxicity of nanocrystalline titanium dioxide, pure and zinc-doped hydroxyapatite nanoparticles in human hepatoma cells. J. Nanoparticle Res..

[cit208] Peng Y., Szalad H., Nikacevic P., Gorni G., Goberna S., Simonelli L. (2023). *et al.*, Co-doped hydroxyapatite as photothermal catalyst for selective CO2 hydrogenation. Appl. Catal. B Environ. Energy.

[cit209] Uskoković V. (2020). Ion-doped hydroxyapatite: an impasse or the road to follow?. Ceram. Int..

[cit210] Ratha I., Datta P., Balla V. K., Nandi S. K., Kundu B. (2021). Effect of doping in hydroxyapatite as coating material on biomedical implants by plasma spraying method: a review. Ceram. Int..

[cit211] Mehrjoo M., Javadpour J., Shokrgozar M. A., Farokhi M., Javadian S., Bonakdar S. (2015). Effect of magnesium substitution on structural and biological properties of synthetic hydroxyapatite powder. Mater. Express.

[cit212] Kaygili O., Keser S. (2015). Sol–gel synthesis and characterization of Sr/Mg, Mg/Zn and Sr/Zn co-doped hydroxyapatites. Mater. Lett..

[cit213] Sprio S., Tampieri A., Landi E., Sandri M., Martorana S., Celotti G. (2008). *et al.*, Physico-chemical properties and solubility behaviour of multi-substituted hydroxyapatite powders containing silicon. Mater. Sci. Eng., C.

[cit214] Predoi D., Iconaru S. L., Predoi M. V., Stan G. E., Buton N. (2019). Synthesis, Characterization, and Antimicrobial Activity of Magnesium-Doped Hydroxyapatite Suspensions. Nanomaterials.

[cit215] Zantye P., Fernandes F., Ramanan S. R., Kowshik M. (2019). Rare Earth Doped Hydroxyapatite Nanoparticles for In vitro Bioimaging Applications. Curr. Phys. Chem..

[cit216] Jose S., Senthilkumar M., Elayaraja K., Haris M., George A., Raj A. D. (2021). *et al.*, Preparation and characterization of Fe doped n-hydroxyapatite for biomedical application. Surf. Interfaces.

[cit217] Iconaru S. L., Predoi M. V., Chapon P., Gaiaschi S., Rokosz K., Raaen S. (2021). *et al.*, Investigation of Spin Coating Cerium-Doped Hydroxyapatite Thin Films with Antifungal Properties. Coatings.

[cit218] De Lima C. O., De Oliveira A. L. M., Chantelle L., Silva Filho E. C., Jaber M., Fonseca M. G. (2021). Zn-doped mesoporous hydroxyapatites and their antimicrobial properties. Colloids Surf., B.

[cit219] Hossain MdS., Tuntun S. M., Bahadur N. M., Ahmed S. (2022). Enhancement of photocatalytic efficacy by exploiting copper doping in nano-hydroxyapatite for degradation of Congo red dye. RSC Adv..

[cit220] Kamieniak J., Bernalte E., Foster C. W., Doyle A. M., Kelly P. J., Banks C. E. (2016). High Yield Synthesis of Hydroxyapatite (HAP) and Palladium Doped HAP via a Wet Chemical Synthetic Route. Catalysts.

[cit221] González-Torres V., Méndez-Sánchez E. R., Gaitan-Cepeda L. A., Torres-Arellano M. E., Díaz-Trujillo G. C. (2014). Characterization and Biocompatibility Evaluation of Hydroxyapatite Doped with Silver and/or Fluorine. Adv. Sci. Technol..

[cit222] Devanand V. G., Ramasamy S., Ramakrishnan V., Kumar J. (2011). Nanocrystalline hydroxyapatite and zinc-doped hydroxyapatite as carrier material for controlled delivery of ciprofloxacin. 3 Biotech..

[cit223] Bharti A., Singh S., Meena V. K., Goyal N. (2016). Structural Characterization of Silver-Hydroxyapatite Nanocomposite: A Bone Repair Biomaterial. Mater. Today Proc..

[cit224] Pang Y., Kong L., Chen D., Yuvaraja G., Mehmood S. (2020). Facilely synthesized cobalt doped hydroxyapatite as hydroxyl promoted peroxymonosulfate activator for degradation of Rhodamine B. J. Hazard. Mater..

[cit225] Tank K., Chudasama K., Thaker V., Joshi M. (2013). Cobalt-doped nanohydroxyapatite: synthesis, characterization, antimicrobial and hemolytic studies. J. Nanoparticle Res..

[cit226] Sebastiammal S., Fathima A. S. L., Henry J., Wadaan M. A., Mahboob S., Wadaan A. M. (2022). *et al.*, Synthesis, Characterization, Antibacterial, Antifungal, Antioxidant, and Anticancer Activities of Nickel-Doped Hydroxyapatite Nanoparticles. Fermentation.

[cit227] Priya B. A., Senthilguru K., Agarwal T., Narayana S. G., Giri S., Pramanik K., Pal K., Banerjee I. (2015). Nickel doped nanohydroxyapatite: vascular endothelial growth factor inducing biomaterial for bone tissue engineering. RSC Adv..

[cit228] Keikhosravani P., Maleki-Ghaleh H., Kahaie Khosrowshahi A., Bodaghi M., Dargahi Z., Kavanlouei M. (2021). *et al.*, Bioactivity and Antibacterial Behaviors of Nanostructured Lithium-Doped Hydroxyapatite for Bone Scaffold Application. Int. J. Mol. Sci..

[cit229] Zhu Z., Yang Y., Fan Y., Zhang L., Tang S., Zhu Y. (2022). *et al.*, Strontium-doped hydroxyapatite as an efficient adsorbent for Cd(II) removal from wastewater: performance, kinetics, and mechanism. Environ. Technol. Innov..

[cit230] Ofudje E. A., Adeogun A. I., Idowu M. A., Kareem S. O. (2019). Synthesis and characterization of Zn-doped hydroxyapatite: scaffold application, antibacterial and bioactivity studies. Heliyon.

[cit231] Chen M. H., Yoshioka T., Ikoma T., Hanagata N., Lin F. H., Tanaka J. (2014). Photoluminescence and doping mechanism of theranostic Eu3+/Fe3+ dual-doped hydroxyapatite nanoparticles. Sci. Technol. Adv. Mater..

[cit232] Nakata K., Kubo T., Numako C., Onoki T., Nakahira A. (2009). Synthesis and Characterization of Silicon-Doped Hydroxyapatite. Mater. Trans..

[cit233] Kalaiselvi V., Loganayaki T., Nathiya M. (2021). Investigation on Pure and Aluminium-doped Hydroxyapatite for Orthopedic Applications. J. Environ. Nanotechnol..

[cit234] Zhao X. Y., Zhu Y. J., Qi C., Chen F., Lu B. Q., Zhao J. (2013). *et al.*, Hierarchical Hollow Hydroxyapatite Microspheres: Microwave-Assisted Rapid Synthesis by Using Pyridoxal-5′-Phosphate as a Phosphorus Source and Application in Drug Delivery. Chem.–Asian J..

[cit235] Byrappa K., Adschiri T. (2007). Hydrothermal technology for nanotechnology. Prog. Cryst. Growth Charact. Mater..

[cit236] Qi Y., Shen J., Jiang Q., Jin B., Chen J., Zhang X. (2015). The morphology control of hydroxyapatite microsphere at high pH values by hydrothermal method. Adv. Powder Technol..

[cit237] Suchanek W. L., Riman R. E. (2006). Hydrothermal Synthesis of Advanced Ceramic Powders. Adv. Sci. Technol..

[cit238] Siddharthan A., Seshadri S. K., Kumar T. S. S. (2006). Influence of microwave power on nanosized hydroxyapatite particles. Scr. Mater..

[cit239] Long B., Li J., Song Y., Du J. (2011). Temperature Dependent Solubility of α-Form l -Glutamic Acid in Selected Organic Solvents: Measurements and Thermodynamic Modeling. Ind. Eng. Chem. Res..

[cit240] Lu L. L., Lu X. Y. (2007). Solubilities of Gallic Acid and Its Esters in Water. J. Chem. Eng. Data.

[cit241] Aliyyu W. C., Riva F. A., Anabel S. M. P., Dwiandhono I., Satrio R., Sari D. N. I. (2024). Nano-hydroxyapatite toothpaste of rice field snail shell combined with basil leaf extract as a remineralizing and antibacterial agent to prevent dental caries. J. Clin.
Exp. Dent..

[cit242] Johnson P., Trybala A., Starov V., Pinfield V. J. (2021). Effect of synthetic surfactants on the environment and the potential for substitution by biosurfactants. Adv. Colloid Interface Sci..

[cit243] Lin D. J., Lin H. L., Haung S. M., Liu S. M., Chen W. C. (2021). Effect of pH on the In vitro Biocompatibility of Surfactant-Assisted Synthesis and Hydrothermal Precipitation of Rod-Shaped Nano-Hydroxyapatite. Polymers.

[cit244] Ren X., Yi Z., Li X. (2024). Novel Synthesis Approach for Natural Tea Polyphenol-Integrated Hydroxyapatite. Pharmaceuticals.

[cit245] Alam M. K., Hossain M. S., Kawsar M., Bahadur N. M., Ahmed S. (2024). Synthesis of nano-hydroxyapatite using emulsion, pyrolysis, combustion, and sonochemical methods and biogenic sources: a review. RSC Adv..

[cit246] Asimeng B. O. , Afeke D. W. and Tiburu E. K., Biomaterial for bone and dental implants: synthesis of B-type carbonated hydroxyapatite from biogenic source, in Biomaterials, 2020

[cit247] Mondal S., Hoang G., Manivasagan P., Moorthy M. S., Kim H. H., Vy Phan T. T. (2019). *et al.*, Comparative characterization of biogenic and chemical synthesized hydroxyapatite biomaterials for potential biomedical application. Mater. Chem. Phys..

[cit248] Abifarin J. K. , Mechanical Reliability and Modeling of Hydroxyapatite Scaffold, Research square, 2021

[cit249] Kawsar M., Hossain M. S., Bahadur N. M., Islam D., Ahmed S. (2025). Crystalline structure modification of hydroxyapatite via a hydrothermal method using different modifiers. Mater. Adv..

